# Modelling the Role of UCH-L1 on Protein Aggregation in Age-Related Neurodegeneration

**DOI:** 10.1371/journal.pone.0013175

**Published:** 2010-10-06

**Authors:** Carole J. Proctor, Paul J. Tangeman, Helen C. Ardley

**Affiliations:** 1 Center for Integrated Systems Biology of Ageing and Nutrition, Institute for Ageing and Health, Newcastle University, Newcastle upon Tyne, United Kingdom; 2 Section of Ophthalmology and Neurosciences, Leeds Institute of Molecular Medicine, St James's University Hospital, Leeds, United Kingdom; Johns Hopkins School of Medicine, United States of America

## Abstract

Overexpression of the de-ubiquitinating enzyme UCH-L1 leads to inclusion formation in response to proteasome impairment. These inclusions contain components of the ubiquitin-proteasome system and α-synuclein confirming that the ubiquitin-proteasome system plays an important role in protein aggregation. The processes involved are very complex and so we have chosen to take a systems biology approach to examine the system whereby we combine mathematical modelling with experiments in an iterative process. The experiments show that cells are very heterogeneous with respect to inclusion formation and so we use stochastic simulation. The model shows that the variability is partly due to stochastic effects but also depends on protein expression levels of UCH-L1 within cells. The model also indicates that the aggregation process can start even before any proteasome inhibition is present, but that proteasome inhibition greatly accelerates aggregation progression. This leads to less efficient protein degradation and hence more aggregation suggesting that there is a vicious cycle. However, proteasome inhibition may not necessarily be the initiating event. Our combined modelling and experimental approach show that stochastic effects play an important role in the aggregation process and could explain the variability in the age of disease onset. Furthermore, our model provides a valuable tool, as it can be easily modified and extended to incorporate new experimental data, test hypotheses and make testable predictions.

## Introduction

UCH-L1 (P09936) is a de-ubiquitinating enzyme (DUB) which binds to small polyubiquitinated proteins and cleaves ubiquitin molecules (P62988). However, as yet its substrates are unknown. It also binds to ubiquitin and stabilises ubiquitin pools [1]. When UCH-L1 is overexpressed it may form dimers which have ubiquitin ligase (E3) activity [2]. E3 ligases bind to specific substrates and accept ubiquitin molecules from ubiquitin-conjugating (E2) enzymes which are then attached to the substrate via an isopeptide bond. UCH-L1 is normally abundant in brain tissue where it is localised to neurons (1-2% of soluble neuronal cell protein) [3]. It is also highly expressed in testes but UCH-L1 levels are low in all other tissues due to silencing by methylation [4]. UCH-L1 expression levels have been associated with several cancer types. It is up-regulated in some cancers; for example it is over-expressed in lung cancer [5]. However there is increased silencing of the UCH-L1 gene in human colorectal and ovarian cancers [6]. UCH-L1 is also found in Lewy bodies of Parkinson's disease (PD) patients and tangles in Alzheimer's disease (AD) patients [7]. It is oxidatively damaged in these diseases and loses about 40–80% of its activity [8]. There is also reduced protein expression of UCH-L1 in PD and AD [9]. This reduction could be due to an increase in damaged UCH-L1 which is then sequestered into inclusions. For example, the level of soluble UCH-L1 protein is inversely proportional to the number of tangles in AD brains [9]. The UCH-L1 gene is also known as PARK5 and mutations in this gene are linked to PD [10], [11]. The I93M mutation has severely diminished hydrolase activity and lower E3 activity compared to WT [2] and it has been suggested that it is linked to disease, although there is controversy regarding this relationship [12]. Recently it was shown that the I93M mutant resembles oxidatively-damaged UCH-L1 in that it has increased insolubility and increased interaction with the Lamp2a (P13473) receptor compared with wild-type UCH-L1 [8], [13], [14]. On the other hand, the S18Y polymorphism may be associated with decreased susceptibility of sporadic PD in a dose-dependent manner, although the evidence for this may be weak [15] or moderate at most [16]. The S18Y mutant has greater hydrolase activity but much lower E3 activity than WT [2], [8], [17]. One of its substrates is α-synuclein but in this case UCH-L1 forms K63-linked ubiquitin chains which are not recognised as a degradation signal by the proteasome. Moreover, the E3 activity of UCH-L1 has only been seen *in vitro* and so far this data has not been replicated.

UCH-L1 is susceptible to oxidative damage and when this occurs it has aberrant functions similar to mutated UCH-L1, as already mentioned. Abnormal UCH-L1 interacts with Lamp2a, Hsc70 (P11142) and Hsp90 (P07900), and may inhibit chaperone mediated autophagy (CMA)-dependent degradation causing CMA substrates, e.g. α-synuclein (P37840) and GAPDH (P04406), to accumulate [13], [14]. UCH-L1 is a long-lived protein with a half-life greater than 48 h and is mainly turned over by macroautophagy [13]. It also accumulates when the proteasome is inhibited suggesting additional turnover by the proteasome [18]. Cellular proteins are susceptible to damage and prone to misfolding. Misfolded proteins may be refolded via the chaperone pathway, or targeted for degradation via the ubiquitin-proteasome pathway. Misfolded proteins may also aggregate and this may happen after the misfolded protein has been targeted for degradation so that ubiquitin and DUBs are sequestered into the aggregate along with the misfolded protein. If the misfolded protein is a UCH-L1 substrate, then UCH-L1 will also end up in aggregates.

α-synuclein is abundantly expressed in human brain (low levels are also found in all other tissues except liver). It is natively unfolded but does not self-aggregate unless it is present at very high levels [19]. However, mutations and oxidative damage can lead to an increase in its propensity to aggregate [20]. It is a fairly stable protein with a half-life of about 16 hours [21], although it has also been shown that its half-life is greater than 19 hours [22]. It has been shown *in vitro* that it can be degraded by the 20S proteasome in a ubiquitin-independent manner [22] and there is also *in vivo* evidence that it is degraded by CMA [23]. If α-synuclein is oxidatively damaged then it is degraded by the ubiquitin-proteasome pathway. For example, the early onset Parkinson's disease associated protein, Parkin (O60260), which has E3 ligase activity, is involved in the polyubiquitination of O-glycosylated α-synuclein [24], [25]. Data suggest α-synuclein is phosphorylated prior to ubiquitination [26]. Parkin also ubiquitinates the α-synuclein-interacting protein, synphilin-1, with both Lys48 and Lys63-linked polyubiquitin chains [27]. However, Lys48 linkages which lead to degradation are only formed when the parkin/synphilin-1 ratio is unusually high.

**Figure 1:**
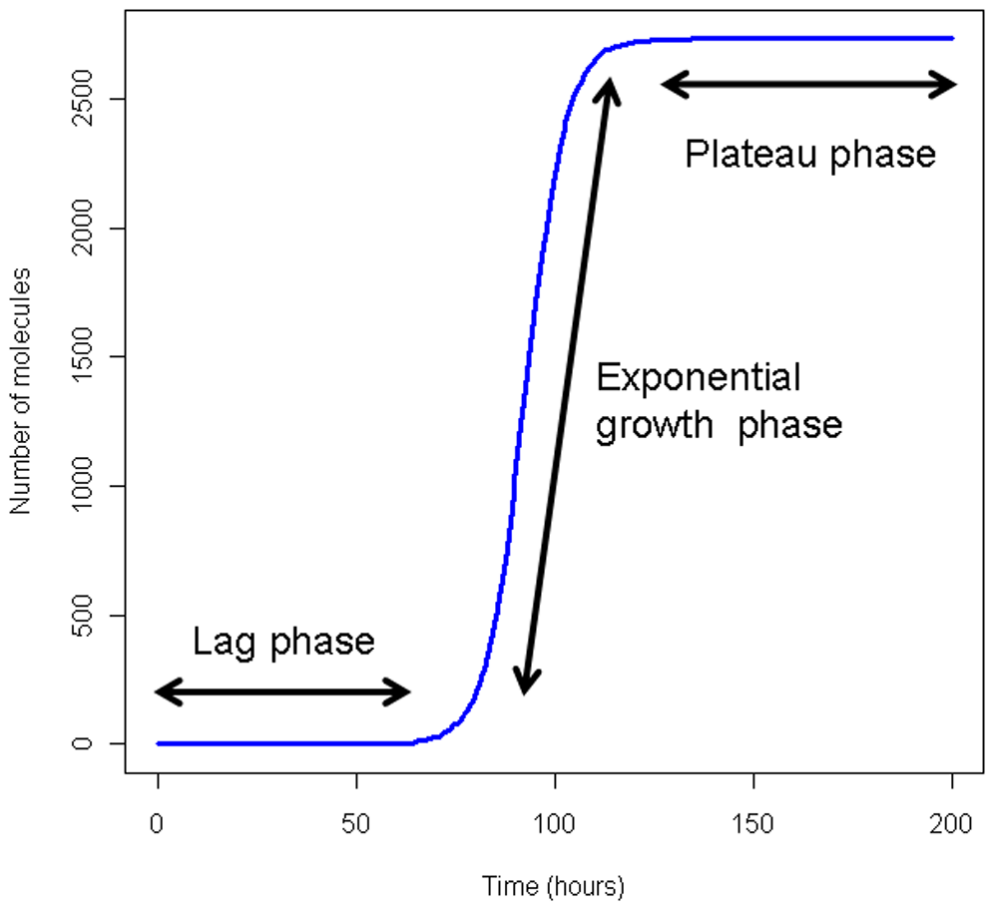
Typical aggregation kinetics. During the lag phase, the aggregation process is reversible and aggregate size remains low. Once a critical size of aggregate is reached, the process is irreversible and the aggregation process is accelerated (exponential growth phase). The pool of aggregatable protein is eventually depleted and the curve reaches a plateau.

There have been many *in vitro* studies of protein aggregation kinetics and many mechanisms and mathematical models have been proposed (see Morris et al 2009 for a recent review) [28]. Although many different schemes for protein aggregation have been proposed, there is a general consensus that there are three main steps. Firstly, the protein monomer has to undergo some modification before it has a propensity to aggregate. Secondly, there is a nucleation step, where monomers interact to form small aggregates. This stage is very slow and also reversible. However, the small aggregates formed at this stage may be toxic to cells as they may interact with cellular components. In particular, they can inhibit the proteasome which is unable to degrade aggregated protein. The third stage begins once the aggregate has reached a certain size, often termed the “nucleus” or the “seed” and is called the elongation stage. At this point, the aggregates irreversibly and rapidly grow in size forming protofilaments and fibrils that are typical in neurodegeneration. Different mechanisms have been proposed for the elongation stage such as the addition of monomers or the assembly of oligomers. Plots of aggregation kinetics typically give rise to a sigmoidal curve which has a lag phase before any aggregation takes place, followed by a sharp increase after nucleation and finally a plateau due to all the protein in the *in vitro* studies being incorporated into the aggregates (see Figure 1). Many examples of aggregation kinetics based on experimental data and a range of deterministic models are shown in a recent review [28].

Based on our previous work [18], we have set up an experimental system to study the kinetics of inclusion formation where UCH-L1 is overexpressed and proteasome activity is inhibited. In order to gain more insights into the processes involved, we have also constructed a stochastic mechanistic model using the Systems Biology Markup Language (SBML) [29]. We used the model to examine the effects of both UCH-L1 expression and proteasome activity on the aggregation process. Since there is controversy over the effects of mutant forms of UCH-L1 we also used the model to investigate the nature of the mutations and then did further experiments to test the model predictions.

## Results

### Normal conditions

**Figure 2:**
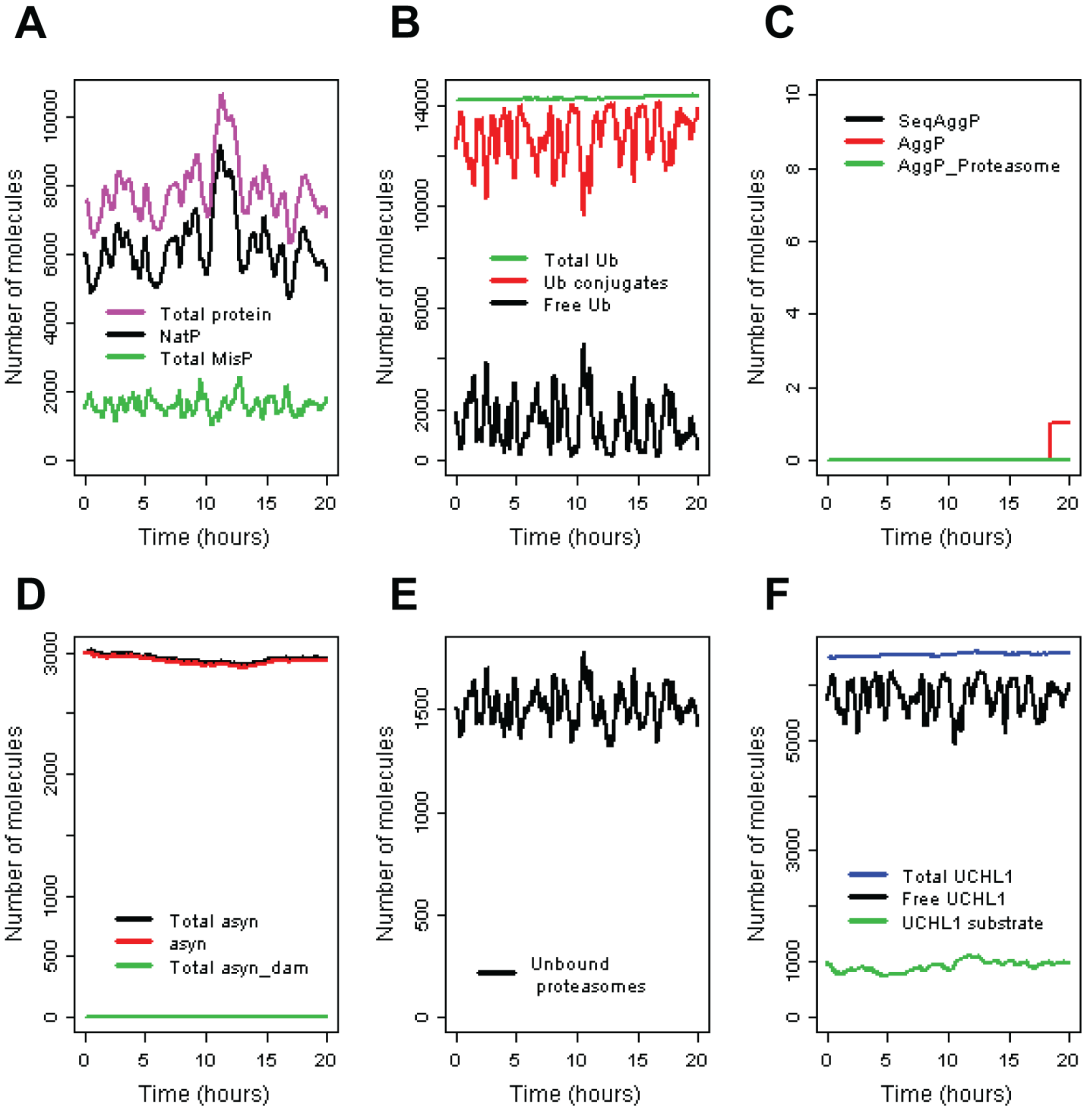
Simulation results for normal conditions. This shows how some of the species in the model vary over time in a typical simulation run. **A** Generic pool of protein. NatP  =  Native protein; TotalMisP =  unbound misfolded protein + all bound forms of misfolded protein; Total protein  =  NatP + TotalMisP. **B** Ubiquitin pools. Ub conjugates includes all complexes containing ubiquitin not just ubiquitinated proteins (e.g. Ub-UCHL1 complex is included in this pool). **C** Aggregated protein. SeqAggP =  aggregates sequestered into inclusion bodies; AggP = small unbound aggregates (of all types); AggP_Proteasome = small aggregates bound to the proteasome. **D** α-synuclein levels: asyn = unbound α-synuclein; asyn_dam = total pool of damaged α-synuclein (except any that is present in inclusions); total asyn = total pool of α-synuclein. **E** Pool of unbound proteasomes. **F** UCHL1 pools: Free UCHL1 = unbound UCHL1; Total UCHL1 = total pool of UCHL1 (except any that is present in inclusions); UCHL1 substrate = total level of the UCHL1 substrate (either bound or unbound).

We adjusted the model parameters so that protein levels remain constant over time with just small fluctuations due to the stochastic component of protein synthesis and degradation (Figure 2). The model predicts that no damage occurs to UCH-L1 or α-synuclein and so no inhibition of CMA takes place. There are low levels of misfolded protein which are targeted for degradation via the ubiquitin-proteasome system and misfolded species remain low so that aggregates rarely form. The mean values of 100 simulations are shown in Figure S1.

### Overexpressing UCH-L1 + proteasome inhibition (PI)

**Figure 3:**
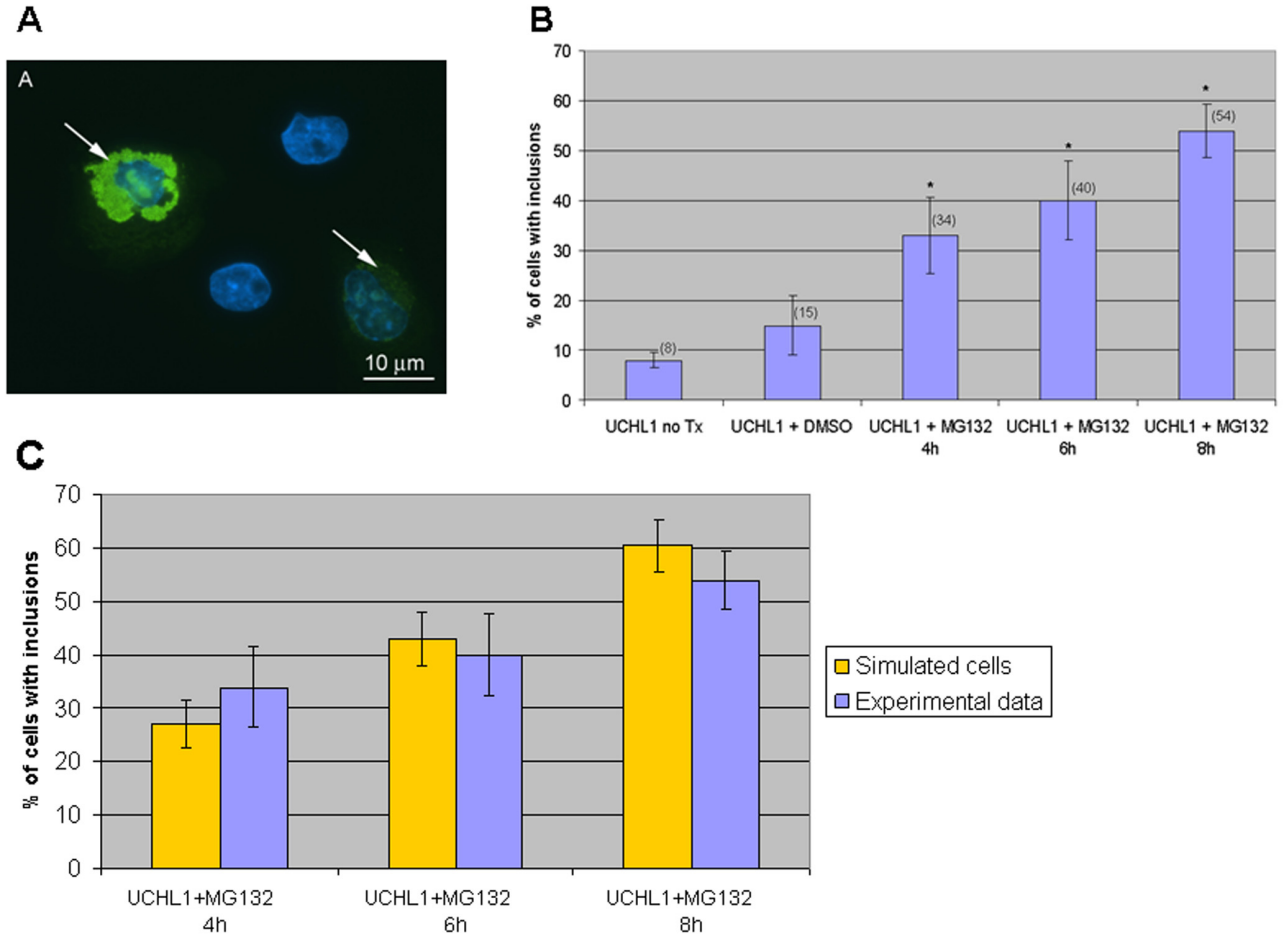
UCH-L1 inclusion formation in COS7 cells over-expressing wild type UCH-L1. Cells were transfected with UCH-L1-HA for a total of 28 h. 5 µM MG132 was added for the times indicated prior to the end of the experiment. **A** Typical example of cells overexpressing UCHL1-HA which contain inclusions. Images immunostained with monoclonal HA (green) and counterstained with DAPI to visualise nuclei (blue). Arrows indicate the position of inclusions within the cell. **B** Time course for UCH-L1 inclusion formation. The presence of UCH-L1 was assessed by immunofluorescence using mouse monoclonal anti-HA antibodies. Bars represent the percentage of cells containing inclusions. Error bars indicate the standard error from the mean of the experiments. Asterisk indicates a significant difference between the percentage of untreated cells versus the percentage of treated cells. *p<0.001. no TX  =  no treatment. **C** Comparison of experimental data and simulation results for the percentage of cells containing inclusions in each experiment. Error bars for simulated data represent the standard error for a percentage (s.e  = 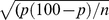, where p is the percentage and n is the number of simulations).

Time course experiments were carried out to look at UCH-L1 inclusions for up to 8 h post addition of 5 µM MG132 in transiently transfected cells overexpressing an UCHL1-HA construct (example of inclusions observed shown in Figure 3A). Figure 3B shows that inclusions can form as early as 4 h but there are significantly more by 8 h (p<0.001). We initially modelled UCH-L1 overexpression by increasing the initial amount of UCH-L1 and the synthesis rate by a factor of 2 and used an SBML event structure to decrease the rate of proteasome degradation by 70% after 20 hours. However, the simulation results showed that there was much less cell variability in the timing of inclusion formation than seen in the experimental data. One possible explanation is that the level of UCH-L1 is not same in each cell. In fact transfection is not 100% efficient with about 25% of cells expressing endogenous levels of UCH-L1 in the UCH-L1-overexpression experiments. Furthermore, some cells may integrate more than one plasmid leading to even higher levels of UCH-L1 than the majority of cells in the experiment. Another possible source of variation is that the expression levels of UCH-L1 may depend on the point of integration of the plasmid into the host DNA. Therefore, simulations were carried out on three sub-populations of cells and then the results combined. We assumed that 25% of simulated cells had endogenous levels of UCH-L1, 50% of simulated cells over-expressed UCH-L1 by a factor of 2, and 25% of simulated cells overexpressed UCH-L1 by a factor of 3. We only counted inclusions that had an amount greater than 20, since very small inclusions are not detectable by immunofluorescence imaging. The simulation results for a total of 200 runs are summarized in Table 1 and the percentage of simulated cells with inclusions has been plotted in Figure 3C with the experimental data for direct comparison. The figure shows that the simulated data compares well with the experimental data both in terms of the mean and the variability. To examine the importance of stochastic effects we also carried out the same procedure using a deterministic simulator. The results are shown in Table 2 and it can be seen that the deterministic solution does not match the data with too few inclusions occurring at early time points and too many inclusions at 8 h post PI. This confirms that as well as the differences between cells in the rate of UCH-L1 production, additional stochastic effects in other cellular processes are required to explain the experimental data.

**Table 1:** Time course of inclusion formation from stochastic model simulations.

	Number of “simulated cells” with inclusions		
UCH-L1 expression(Number of runs)	4 h post PI	6 h post PI	8 h post PI
3x baseline (50)	37	47	50
2x baseline (100)	16	38	70
baseline (50)	1	1	1
**Total (200)**	**54**	**86**	**121**
**% of simulated cells**	**27**	**43**	**60.5**
**Experimental data**	**34**	**40**	**54**

**Table 2:** Time course of inclusion formation from deterministic model simulations.

	Number of “simulated cells” with inclusions		
UCH-L1 expression(Number of runs)	4 h post PI	6 h post PI	8 h post PI
3x baseline (25)	25	25	25
2x baseline (50)	0	0	50
baseline (25)	0	0	0
**Total (100)**	**25**	**25**	**75**
**% of simulated cells**	**25**	**25**	**75**
**Experimental data**	**34**	**40**	**54**

**Figure 4:**
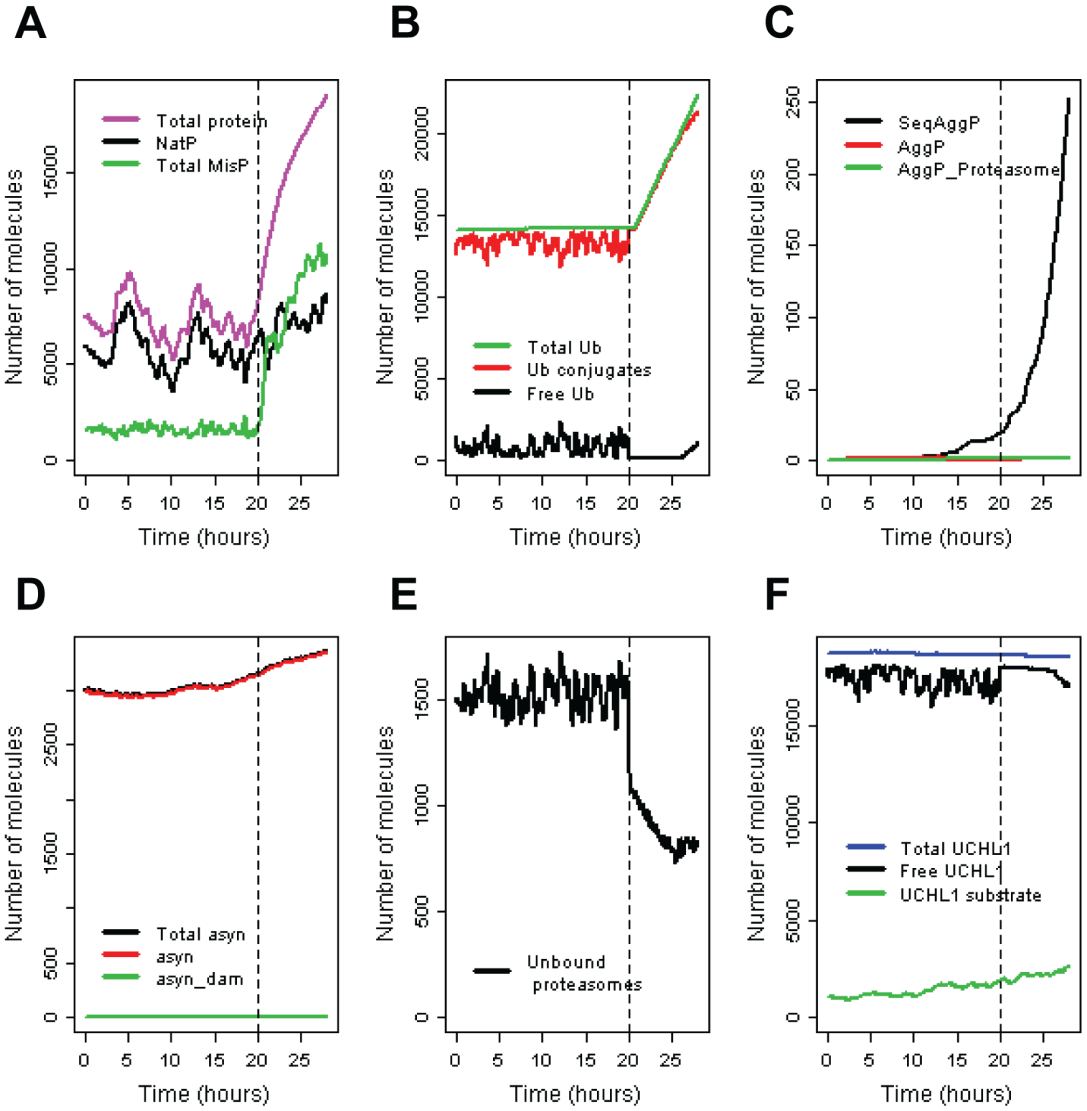
Overexpression of UCH-L1 (3x baseline) with PI at 20 hours (shown by vertical dashed line). **A** Generic pool of protein. NatP  =  Native protein; TotalMisP =  unbound misfolded protein + all bound forms of misfolded protein; Total protein  =  NatP + TotalMisP. **B** Ubiquitin pools. Ub conjugates includes all complexes containing ubiquitin not just ubiquitinated proteins (e.g. Ub-UCHL1 complex is included in this pool). **C** Aggregated protein. SeqAggP =  aggregates sequestered into inclusion bodies; AggP = small unbound aggregates (of all types); AggP_Proteasome = small aggregates bound to the proteasome. **D** α-synuclein levels: asyn = unbound α-synuclein; asyn_dam = total pool of damaged α-synuclein (except any that is present in inclusions); total asyn = total pool of α-synuclein. **E** Pool of unbound proteasomes. **F** UCHL1 pools: Free UCHL1 = unbound UCHL1; Total UCHL1 = total pool of UCHL1 (except any that is present in inclusions); UCHL1 substrate = total level of the UCHL1 substrate (either bound or unbound).

The model predictions for a typical simulated cell with high levels of UCH-L1 (3x baseline) are shown in Figure 4. Note that where total pools of specific proteins are plotted, these totals do not include any protein which is present in inclusions. In this simulation inclusions begin to form at 15 hours, which was 5 hours before proteasome inhibition took place. This is due to very high levels of UCH-L1 which lead to an increase in the UCH-L1 substrate and also more damaged UCH-L1. The increase in damaged UCH-L1 particularly has an effect on the aggregation process as not only is it possible for UCH-L1 to self-aggregate but it also binds to Lamp2a receptors and inhibits α-synuclein degradation. However, during the first few hours, levels of inclusions (SeqAggP) are very low and probably undetectable. The model predicts that 27% of cells have inclusions by 24 hours (4 h post-PI) and this increases to 60.5% by 28 hours (8 h post-PI). The inclusions contain α-synuclein, UCH-L1, ubiquitin, misfolded protein, E3 ligase and the generic DUB. However there is very little damage to α-synuclein and no Parkin is contained in the inclusions generated by this model.

The effects of UCH-L1 overexpression alone can be examined by studying the graphs before the time at which proteasome inhibition begins (up to 20 hours). Ubiquitin pools show a very slight increase from the start of the simulation due to increased stabilization by UCH-L1. The UCH-L1 substrate increases with time, since UCH-L1 overexpression leads to increase DUB activity of its substrate which has the effect of lowering the degradation rate. When inclusions start to form, the substrate may be sequestered into the inclusion and so levels of the substrate increase more slowly and may eventually level off or even start to decrease. The substrate may be bound by ubiquitin and UCH-L1 when it is sequestered into inclusions.

**Figure 5:**
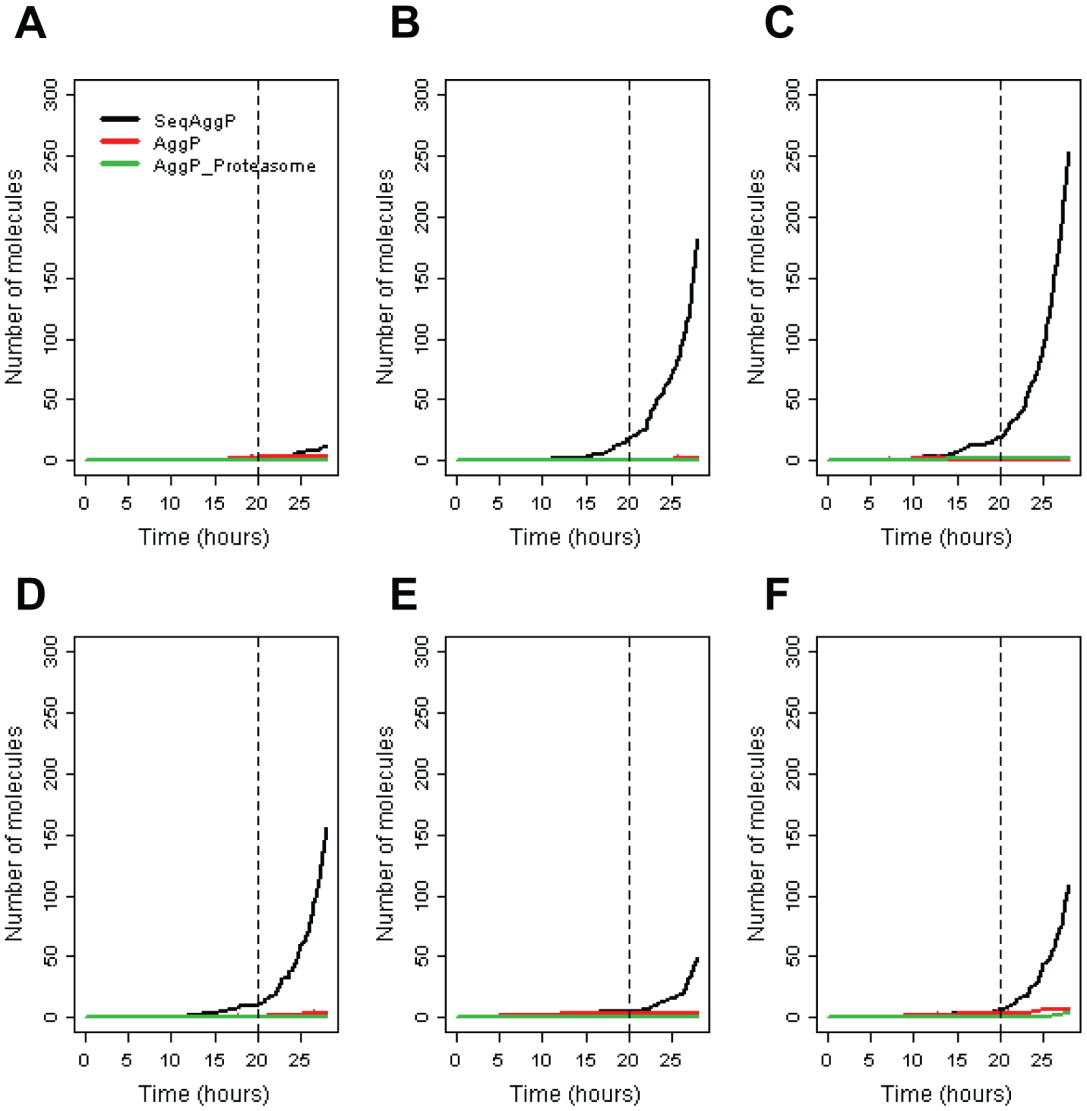
A–F Six simulation runs showing variability in aggregation kinetics when UCH-L1 is overexpressed (x3) and proteasome is inhibited at time t = 20 h (shown by vertical dashed line). SeqAggP =  aggregates sequestered into inclusion bodies; AggP = small unbound aggregates (of all types); AggP_Proteasome = small aggregates bound to the proteasome.

Overexpression of UCH-L1 does not affect levels of the generic protein (NatP) before PI. After PI occurs, levels of NatP start to increase due to less degradation via the proteasome. When inclusions form, misfolded protein may be sequestered into the inclusion and so total protein levels increase less steeply. The effect on α-synuclein varied between simulation runs, although there was usually a slight increase in levels before PI, in some runs an increase was not seen until after PI at which point the increase was continued steeply until inclusions were formed. Since the time at which inclusions starts to form is very variable, we plotted the aggregation kinetics for six different simulations runs where UCH-L1 was overexpressed by a factor of 3 (Figure 5). It can be seen that in some cases (about 10%), inclusions reach levels of detection before the proteasome is inhibited, although the rapid increase in inclusion size only occurs after proteasome inhibition. In simulations where UCH-L1 was overexpressed by a factor of two, this happened rarely (<1% of simulated cells). We also ran the model with UCH-L1 overexpressed three-fold without proteasome inhibition to check if inclusion formation was simply due to UCH-L1 overexpression. In this case, the model predicts that inclusions do start to form between 15 and 22 hours but growth is much slower and in the majority of cells only reach very low levels by 28 h (data not shown). This confirms that proteasome inhibition greatly speeds up the aggregation process.

In order to identify the event or chain of events that leads to inclusion formation we ran the model using a deterministic simulator and made a careful comparison of the results with the stochastic output. We examined the time course of the different species in the model making a careful distinction of events that occurred before and after proteasome inhibition. We used the model with UCH-L1 overexpressed by a factor of 2 with proteasome inhibition at 20 hours for this analysis. Our model output shows that the first event in the chain is an increase in levels of damaged UCH-L1 which binds to Lamp2a receptors leading to an increase in α-synuclein levels. The levels of α-synuclein are now sufficient to form small aggregates and the deterministic solution shows that on average a cell would have formed a small inclusion (the seed required for the exponential phase to begin) before the proteasome is inhibited. After proteasome inhibition there is a large increase in misfolded protein which then gets sequestered into the inclusion and so it rapidly grows in size. In the stochastic model, the seed may not form until after the proteasome is inhibited and so some cells do not have detectable inclusions even by 8 h post PI whereas the deterministic model predicts all cells to have inclusions by 7.2 h post PI.

We assumed that 25% of cells were not transfected and that 25% of cells incorporated more than one extra copy of the plasmid. It is not easy to measure the exact proportions and so we examined the effects of varying these proportions in our model. We found that there was little effect on the model predictions for the proportions of cells containing inclusions by 8 hours after proteasome inhibition which was always in the range of 56–62%. However, lowering the proportion of cells with an extra copy of the plasmid, led to fewer inclusions at 4 hours post PI with only 10% of cells containing inclusions if in the extreme case we assumed that no cells incorporated an extra plasmid. An alternative way to model different expression levels of UCH-L1 would be to use random numbers to decide on the proportions of cells incorporating one extra plasmid or no plasmid. This would provide a more stochastic approach to the problem although we would not expect the model predictions to be greatly affected.

### Mutant forms of UCH-L1

#### I93M mutation

**Figure 6:**
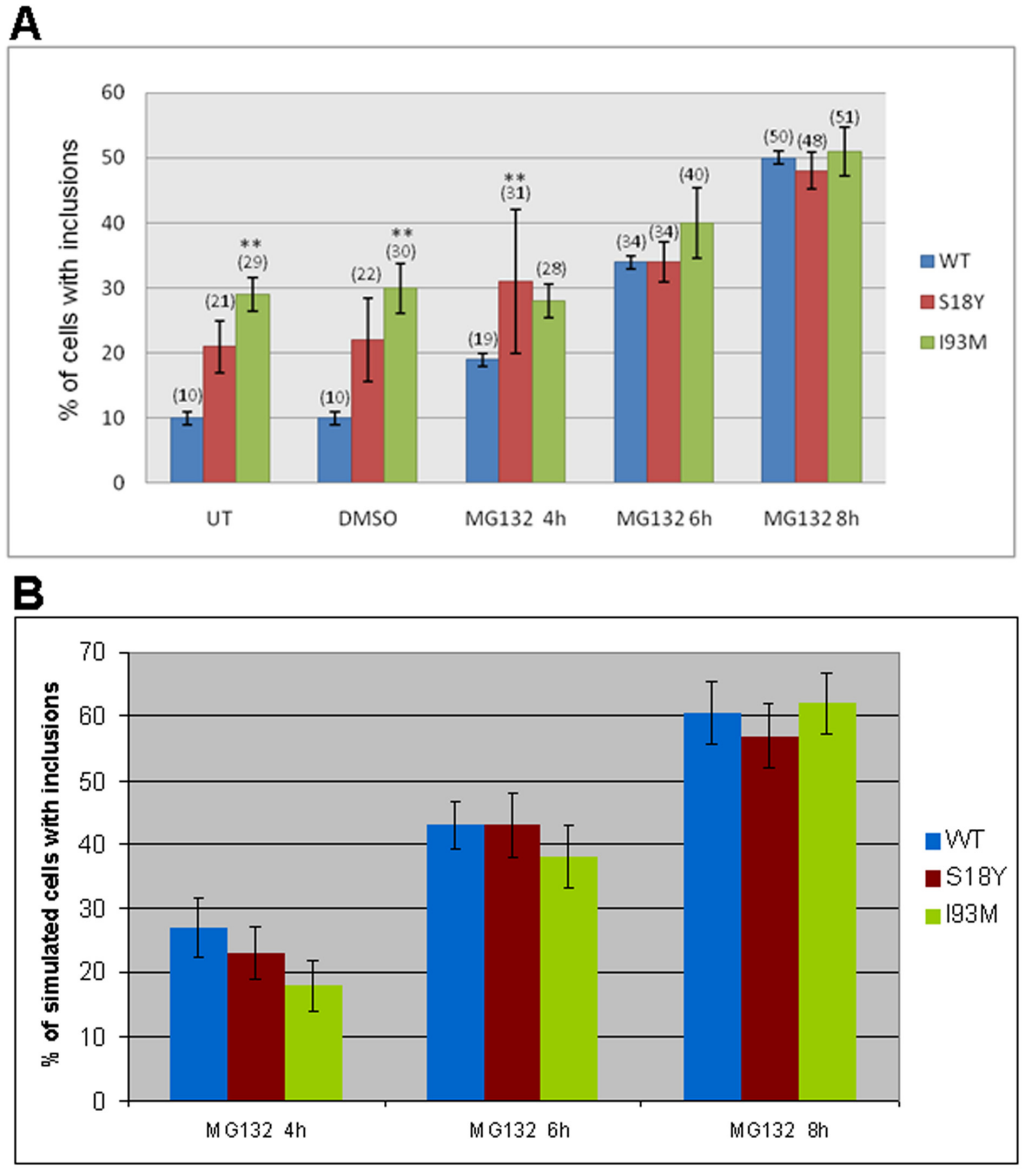
UCH-L1 inclusion formation in COS7 cells over-expressing mutant forms of UCH-L1. **A** Cells were transfected with wild type or mutant UCH-L1 HA tagged plasmids for a total of 28 h. 5 µM MG132 was added for the times indicated prior to the end of the experiment. Time course for formation. The presence of UCH-L1 was assessed by immunofluorescence using polyclonal UCH-L1 antibodies. Bars represent the percentage of cells containing inclusions. Error bars indicate the standard error from the mean of the experiments. Asterisk indicates a significant difference between the percentage of untreated cells versus the percentage of treated cells. **p<0.001. no TX  =  no treatment. **B** Percentage of simulated cells with inclusions for wild-type or mutant UCHL-L1. Error bars indicate standard error for a percentage (s.e  = 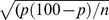, where p is the percentage and n is the number of simulations).

We used the model to examine the possible effects of UCH-L1 mutations on the aggregation process. To mimic the effect of the I93M mutation we first assumed that it had 55% lower hydrolase activity than wild-type UCH-L1 [8]. The simplest way to model this was to adjust the parameter *k_actUCHL1_* to reflect the relative amount of wild-type and I93M UCH-L1 in the cell. Our model predicted that there was a small increase in the number of inclusions at 4 h, 6 h, and 8 h after proteasome inhibition but the difference was only significant at 6 h (p = 0.04). The reason for the slight increase in inclusion is that more of the UCH-L1 substrate ends up at the proteasome but is degraded much less efficiently and is more likely to be sequestered into inclusions. We then modified the model to allow for the possibility that the I93M mutant has lower solubility than wild-type UCH-L1 and has similar properties to oxidatively damaged UCH-L1 in addition to having lower hydrolase activity. We modelled this by including an additional species UCHL1_mut to represent the I93M mutant and assumed that this had a higher propensity to become damaged. If we assumed that the rate of damage was an order of magnitude higher, then the model predicted that inclusions containing high levels of α-synuclein and UCH-L1 started to form before proteasome inhibition with 75% of cells containing inclusions by 4 hours after proteasome inhibition with only the cells that we had assumed were not transfected without inclusions. If the rate of damage for mutant UCH-L1 was 50% higher, then the model predicted that 18% cells contained inclusions by 4 hours after proteasome inhibition, rising to about 62% by 8 hours post PI. We also ran the model without proteasome inhibition for 28 hours and in this case, 31% of cells formed inclusions by the end of the simulations. We also performed experiments in the laboratory to test the model predictions and found that the results were in close agreement with the model which assumed that the I93M mutant had a 50% greater chance of being damaged (Figure 6 and Table S9).

#### S18Y mutation

It has been suggested that the S18Y mutant has slightly higher hydrolase activity than wild-type UCH-L1 so we modelled this mutation by increasing UCH-L1 activity by 20%. The model predicts that the numberof inclusions is very similar to the model with wild-type UCH-L1 (Figure 6 and compare Table 1 and Table S10). Our laboratory experiments show that more inclusions are observed in untreated cells and those treated with MG132 for 4 hours with similar levels of inclusions to wild-type for all other time-points (Figure 6) which indicates that the S18Y mutation is not protective in our system. This suggests that the S18Y mutant may also behave like damaged UCH-L1. Therefore, it would be desirable to examine the binding affinity of S18Y mutant to Lamp2a receptors in order to shed some light on why more inclusions are observed when SI8Y is over-expressed. It may be that the beneficial effects of S18Y are only observed when total levels of UCH-L1 are close to basal levels.

## Discussion

We have extended our generic ubiquitin-proteasome model [30] to include turnover of α-synuclein, UCH-L1, and a substrate of UCH-L1. We also modelled damage and aggregation of these proteins. During the model simulations we can keep track of which proteins end up in inclusions by using dummy species as products in the reactions for inclusion formation. We found that the main components were α-synuclein, ubiquitin, damaged UCH-L1 and misfolded protein. Due to the high levels of ubiquitin present, we assume that most of the misfolded protein present was ubiquitinated. There was also the generic DUB and E3 ligase present in inclusions indicating that the failure of the proteasome system was responsible for many of the proteins present in the inclusions. These proteins are also found in inclusions in laboratory experiments [e.g. 18].

We conducted laboratory experiments to further examine the effects of UCH-L1 overexpression and proteasome inhibition on the aggregation process based on our previous work [18]. Our model showed that the large variability in aggregation kinetics seen in the experimental system could not be explained by stochastic effects alone if all cells were identical in respect to the amounts of proteins that they contained. However, there is intracellular variability in the expression of UCH-L1 in the experiments and so we accounted for this in the model by running sets of simulations with different levels of UCH-L1. In this case, the model predictions give a close match to the experimental data. We found that in some simulations, inclusions begin to form even before proteasome inhibition, when UCH-L1 is overexpressed by a factor of three. This was due to increased levels of damaged UCH-L1 and an increase in α-synuclein pools. Therefore we ran simulations without proteasome inhibition to see if high over-expression of UCH-L1 alone could lead to inclusions. The model predicts that although inclusions form by 28 h in the majority of cells, levels are much lower and we do not see the exponential increase at 20 h as was the case when the proteasome is inhibited (data not shown). So we conclude that proteasome inhibition greatly speeds up the aggregation process. This is not surprising considering the wealth of evidence for the role of proteasome impairment in neurodegeneration [22], [31], [32], [33], [34], [35], [36], [37].

We used the model to examine the effect of UCH-L1 mutations and then carried out further experiments to test the model predictions. For the I93M mutation we assumed that either the mutant had lower hydrolase activity than wild-type or that in addition the I93M mutant had a greater propensity to be damaged. We found that the experimental data did not agree with the model predictions if the only effect of UCH-L1 was reduced hydrolase activity. We found that the closest agreement between model predictions and data required that the I93M mutant had a damage rate that is about 50% higher than wild-type.

We also noted that the results of our short term experiments revealed differences to our previous longer term ones [31]. The earlier experiments were carried out for 44 h and we found that the percentage of cells with inclusions without inhibitors was similar for wild-type and S18Y mutants, but with MG132 both S18Y and I93M had higher levels of inclusions than wild-type. It may be that in longer term transfections the over expressing cells with S18Y are capable of clearing some of the naturally occurring inclusions. Conversely, elevated levels of inclusions observed with I93M without inhibitor in our earlier work may be caused due to increased sensitivity of the cells to over expression of mutant protein. This suggests that prolonged expression of mutant protein is required in order to see effects. This is not dissimilar to what seen in disease, as familial patients survive several decades before symptoms present [11].

Mitochondrial dysfunction has been linked to neurodegenerative diseases and the evidence is especially strong in Parkinson's disease. Parkin plays a key role in mitochondrial dynamics and is linked to the PTEN-induced putative kinase 1 protein, PINK1 (Q9BXM7) (reviewed in [38]). There is also recent evidence to suggest that Parkin and PINK1 act together to maintain mitochondrial homeostasis by targeting dysfunctional mitochondria for mitophagy [39]. We are currently developing a model of mitochondrial dynamics as it is our intention to eventually develop an integrative model of the network involved in preventing protein aggregation.

**Figure 7:**
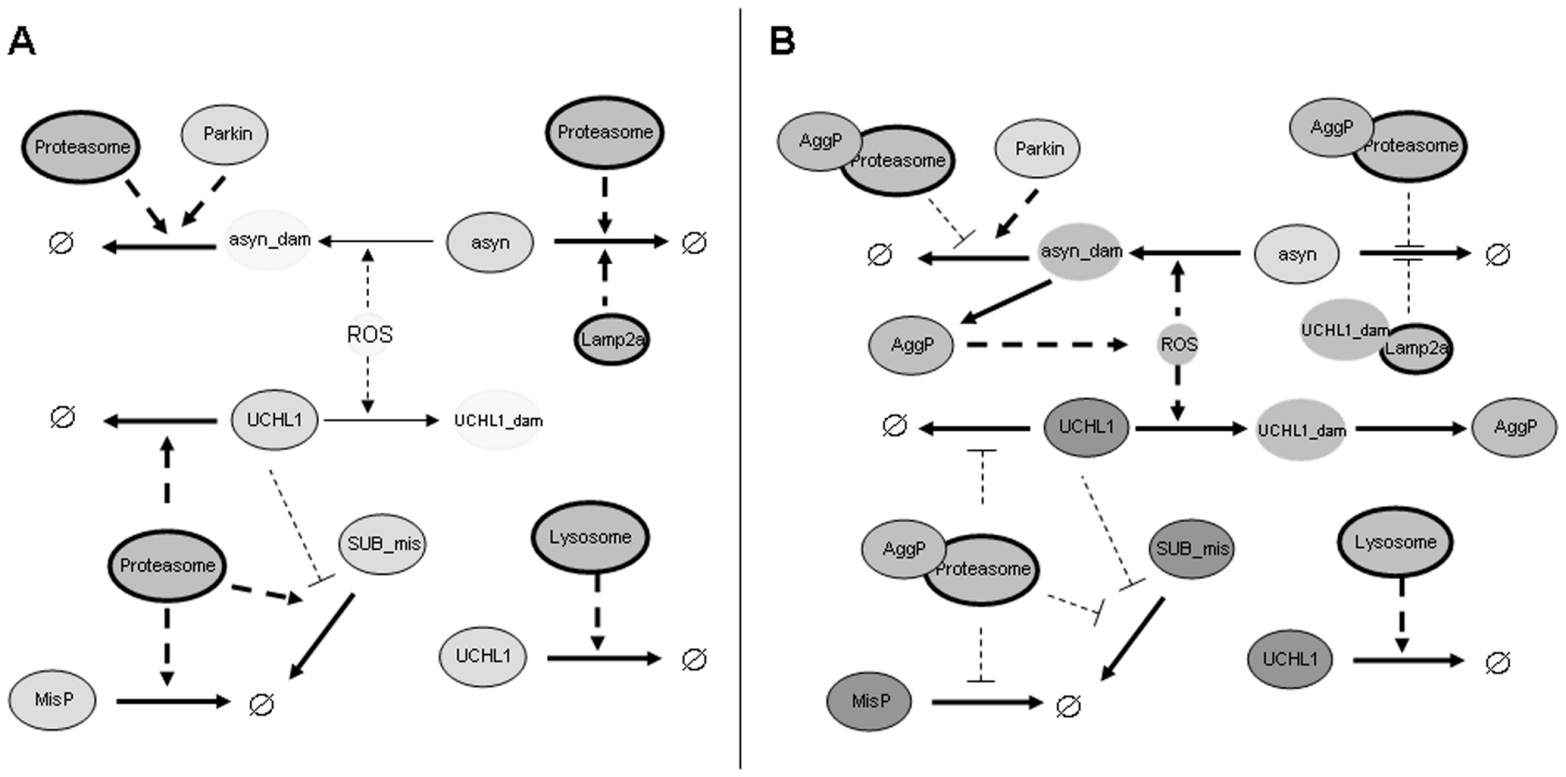
The involvement of UCH-L1 in exacerbating any disturbances in protein homeostasis: A under normal conditions, damaged proteins are cleared by proteasomes or lysosomes and there is no protein aggregation; B when proteasomes and/or lysosomes become inhibited, UCH-L1 levels rise and the pool of damaged UCH-L1 also increases. This leads to inhibition of chaperone-mediated autophagy and also less degradation of UCH-L1 substrates via the ubiquitin-proteasome system. As a result protein aggregates start to form leading to further proteasome inhibition and increased levels of oxidative stress. The grey scale indicates the abundance of each species with white representing very low levels and dark grey representing high levels. Degradation is represented by the empty set symbol (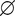).

It is known that proteasome efficiency declines with age and that this can result in an accumulation of damaged and misfolded proteins [40]. There is also a decline in lysosomal degradation due to an increase in lipofuscin and dysfunctional mitochondria [41], [42], [43]. In addition, there is evidence that there is crosstalk between proteasomal and lysosomal pathways and inhibition of one pathway ultimately leads to inhibition of the other probably due to an overload of the systems [44], [45]. This will lead to an increase in protein aggregates which themselves inhibit the proteasome and may also increase levels of reactive oxygen species so that a vicious cycle might ensue. The decrease in protein degradation will also lead to an increase in the pools of proteins especially those with short half-lives unless protein synthesis is also downregulated. In tissues which express UCH-L1, a gradual increase in levels of this protein with age may be particularly problematic since it is particularly susceptible to oxidative damage and subsequently inhibits chaperone-mediated autophagy. Figure 7 illustrates the effects of a disturbance in protein homeostasis: (A) under normal conditions, damaged proteins are cleared by proteasomes or lysosomes and there is no protein aggregation; (B) when proteasomes and/or lysosomes become inhibited, UCH-L1 levels rise and the pool of damaged UCH-L1 also increases. This leads to inhibition of chaperone-mediated autophagy and also less degradation of UCH-L1 substrates via the ubiquitin-proteasome system. As a result protein aggregates start to form leading to further proteasome inhibition and increased levels of oxidative stress. The initiating event in this vicious cycle could be one of several candidates. For example, it could be an increase in oxidative stress which leads to an increase in damaged protein; an increase in UCH-L1 pools due to decreased degradation; or inhibition of lysosomal pathways due to lipofuscin or dysfunctional mitochondria. It is likely that stochastic factors will also play an important role since the actual components damaged will have a large effect on subsequent outcomes. The timing of events will also be highly dependent on stochastic effects and could explain why there is large variability in the age of onset of disease. Our experimental system and model can be used to examine the changes that occur during ageing and age-related disease by accelerating the processes involved.

## Materials and Methods

### Building the model

We extended our earlier model of the ubiquitin-proteasome system [30] to include detail of UCH-L1 and α-synuclein. The original model was encoded in the SBML [29] so that it was straight-forward to modify and extend. There are many freely available tools for building and modify SBML models (www.sbml.org). We used SBML shorthand and a Python script to convert the code into full SBML [46]. Since there is large variability in biological data, we have built stochastic models, and simulations are run using the Gillespie algorithm [47] on the BASIS (Biology of Ageing e-Science Integration and Simulation) system [48], [49]. The model is freely available from the BASIS website (www.basis.ncl.ac.uk) and the Biomodels database [50], [51] (ID:MODEL0912070000). The SBML code is also available in the supporting information (Dataset S1).

The model is quite large but can be split up into eight components: a generic model of the ubiquitin-proteasome system; UCH-L1 turnover, UCH-L1 stabilisation of ubiquitin pools; UCH-L1 hydrolase activity, UCH-L1 damage; α-synuclein turnover, α-synuclein damage; and protein aggregation. Each component is described in turn and each individual component is itself a complete model which can be tested. The components are then linked together to form an integrative model. Full details of the model species and reactions are given in the supporting information in Tables S1, S2, S3 and S4. Further details of the parameter values are also described in the supporting information (Supporting Text S1).

### Generic model of the ubiquitin-proteasome system

This component is based on the already published model of the ubiquitin-proteasome system [30]. The original model assumed that total ubiquitin pools are constant. However, ubiquitin is upregulated after stress and ubiquitin itself is turned over by proteasomes and so the model has been slightly modified to include ubiquitin turnover. This required the addition of three reactions: basal synthesis, degradation and stress-induced synthesis of ubiquitin (Table S5). We assume that the rate of stress-induced synthesis depends on the level of misfolded protein in the cell.

### UCH-L1 turnover

We assume that UCH-L1 can be degraded by lysosomes and proteasomes. This component consists of just four reactions: synthesis, binding to the proteasome, degradation by the proteasome and degradation by lysosomes (Table S6).

### Regulation of monomeric ubiquitin pools

UCH-L1 binds to ubiquitin (by monoubiquitination) and prevents ubiquitin (Ub) degradation. This is reversible, so when pools of Ub are low, Ub is released from UCH-L1. This enables Ub levels to increase rapidly before upregulation takes place which is subject to a delay due to the time required for protein synthesis. When Ub pools are sufficient again, Ub can bind to UCH-L1. We assume that the affinity of UCH-L1 to Ub is less than affinity of Ub to E1 and E2 enzymes, so that it only binds surplus pools (Table S6).

### Hydrolase activity

The substrates of UCH-L1 are currently unknown so we include a pool of a generic substrate named SUB. We assume that UCH-L1 first binds (reversibly) to the ubiquitinated (or polyubiquitinated) substrate and removes Ub processively (Table S6).

### Damage of UCH-L1

We assume that UCH-L1 may be oxidatively damaged by reactive oxygen species (ROS (CHEBI:26523)) which causes it to lose its activity. We assume that damaged UCH-L1 will bind to the Lamp2a receptor which prevents binding of CMA substrates to the receptor (Table S6).

### α-synuclein turnover

We assume that α-synuclein is degraded by the proteasome without the need for ubiquitination (only mutated forms require ubiquitination), or chaperone-mediated autophagy which requires binding of α-synuclein to the Lamp2a receptor (Table S7).

### α-synuclein damage

We assume that α-synuclein may be damaged by ROS and it is then ubiquitinated by Parkin and degraded via the 26S proteasome (Table S7).

### Aggregation of protein

We assume that any misfolded protein can interact with another misfolded protein to form a small aggregate. An aggregate can grow in size by the addition of further misfolded proteins and may decrease in size by the removal of misfolded proteins with the aid of the chaperone machinery. However, when the aggregate reaches a certain threshold size, disaggregation can no longer take place and instead an inclusion forms. This threshold represents the seed and is assumed to be of size six based on data for amyloid fibril polymerization [52]. For simplicity we assume that misfolded species only interact with other species that are of the same type, i.e. α-synuclein may interact with itself but not with a generic misfolded protein. The model contains 5 different species which may aggregate: generic misfolded protein; a substrate of UCH-L1 in its misfolded state; native α-synuclein; damaged α-synuclein; and damaged UCH-L1. We assume that damaged protein has a greater propensity to aggregate than native protein. Once an inclusion forms, any misfolded or damaged protein may be sequestered even if it is ubiquitinated or bound to E3 ligases although the rate of this happening is normally very low. This must occur since E3 ligases, chaperones and ubiquitin are found in inclusion bodies [18]. The circumstances in which this would occur is when the ubiquitin proteasome system is overwhelmed and degradation slows down so that these misfolded proteins persist for longer time periods and therefore are more likely to be sequestered into inclusions. The larger the amount of inclusions in the cell, the more likely they are to increase in size and so inclusion growth may be very rapid after cellular stress. We assume that inclusions are a protective mechanism and that they do not interfere with the cellular machinery. On the other hand, we assume that small aggregates are detrimental since they may bind to the proteasome and inhibit proteasomal activity and that they may also cause an increase in ROS production. The reactions for aggregation are listed in the supporting information (Tables S5, S6 and S7).

We carried out a sensitivity analysis to see which parameters affected the kinetics of aggregate formation. The results are reported in Table S8 and discussed in the supporting information (Supporting Text S1).

### Experimental procedures

#### Early time course experiments to look at UCH-L1 inclusions

COS7 cells were transfected with 2 µg UCHL1-HA, S18Y-HA or I93M-HA plasmid DNA in 2% FCS containing media as previously described [18]. Cells were untreated, treated with the carrier DMSO or treated with 5 µM MG132 for 4, 6 or 8 h and the experiment was terminated at 28 h post transfection. Cells were processed for immunofluorescence using monoclonal 262K HA antibody (Cell Signalling Technologies) or polyclonal anti-UCHL1 (PGP 9.5, Biomol) as previously described [18]. A Nikon Eclipse TE-2000-E microscope was used to observe the number of cells containing inclusions A minimum of 300 transfected cells were counted from blinded samples. This was repeated at least 2 or 3 times. It was not possible to count the actual number of inclusions per cell and so cells were scored either as containing inclusions or having no inclusions. Then the percentage of cells with inclusions was calculated for each experiment. Data was processed by one way Anova (Bonferroni) statistical analysis using SSPS 16.0 for Windows. Images were captured using Velocity 5 (Improvision) software.

## Supporting Information

Text S1 Further details of the model and simulation methods.
(0.06 MB DOC)

Figure S1 Plot of mean values for 100 runs of model under normal conditions. A Generic pool of protein. NatP  =  Native protein; TotalMisP =  unbound misfolded protein + all bound forms of misfolded protein; Total protein  =  NatP + TotalMisP. B Ubiquitin pools. Ub conjugates includes all complexes containing ubiquitin not just ubiquitinated proteins (e.g. Ub-UCHL1 complex is included in this pool). C Aggregated protein. SeqAggP =  aggregates sequestered into inclusion bodies; AggP = small unbound aggregates (of all types); AggP_Proteasome = small aggregates bound to the proteasome. D α-synuclein levels: asyn = unbound α-synuclein; asyn_dam = total pool of damaged α-synuclein (except any that is present in inclusions); total asyn = total pool of α-synuclein. E Pool of unbound proteasomes. F UCHL1 pools: Free UCHL1 = unbound UCHL1; Total UCHL1 = total pool of UCHL1 (except any that is present in inclusions); UCHL1 substrate = total level of the UCHL1 substrate (either bound or unbound)
(0.17 MB TIF)

Table S1 Model species for generic UPS component.
(0.05 MB DOC)

Table S2 Model species for UCHL1 turnover, activity, damage and aggregation.
(0.04 MB DOC)

Table S3 Model species for α-synuclein turnover, damage and aggregation.
(0.04 MB DOC)

Table S4 Dummy species used to identify different proteins in inclusions.
(0.03 MB DOC)

Table S5 Reactions for generic UPS component.
(0.07 MB DOC)

Table S6 Reactions for UCH-L1 turnover, activity, damage and aggregation.
(0.06 MB DOC)

Table S7 Reactions for α-synuclein turnover, damage and aggregation.
(0.06 MB DOC)

Table S8 List of parameters which have an effect on inclusion formation.
(0.03 MB DOC)

Table S9 Results for I93M mutant. Mutant has lower hydrolase activity and has 50% higher damage rate than wild-type.
(0.03 MB DOC)

Table S10 Results for S18Y mutant. Mutant has higher hydrolase activity.
(0.03 MB DOC)

Dataset S1 SBML code of model for normal conditions.
(0.01 MB TAR)
