# Supplementary material for: Modelling the Role of UCH-L1 on Protein Aggregation in Age-Related Neurodegeneration
Source: PLoS One. 2010 Oct 6;5(10):e13175. doi: 10.1371/journal.pone.0013175 (PMC2950841; doi:10.1371/journal.pone.0013175)
Supplement: Text S1 — Further details of the model and simulation methods. (0.06 MB DOC) [file pone.0013175.s001.doc]

**Further details of the model**

**Model species and initial amounts**

We chose the initial amounts for each species based on our previous models. It is not feasible to use the total amounts of each protein that would be found in cells as these numbers are too large for stochastic simulation. There is no detailed information about the actual amounts of many of the model species available in the literature and so we have to make some assumptions about the relative amounts of each protein. For example, experimental data in IMR-90 cells show that there are approximately 10^7^ molecules of ubiquitin per cell [1]. Since we are only modelling part of the total cellular protein, we have scaled down the level of total ubiquitin to 14000 molecules per cell. The total level of proteasomes per cell has been estimated to be 8 × 10^5^ per cell in L929 cells [2] which gives a ratio of about one proteasome to every 10 ubiquitin molecules. Since ubiquitin is involved in other processes apart from protein degradation we chose a ratio of about one proteasome to every 7 ubiquitin molecules and set the initial total of proteasomes equal to about 1900.

We ran 100 simulations of the model for normal conditions and plotted the totals of each protein in the model over time. In some cases, such as pools of free ubiquitin and ubiquitin conjugates, the levels did not reach steady state until after 4 hours of simulation time. Therefore we calculated the average value of each species at the end of the simulation and then used these values as the initial amounts. This accounts for why we chose to start off with some misfolded and ubiquitinated protein and proteins bound by the proteasome and DUBs (including UCH-L1) at the start of the simulation. The initial amounts of all the species are shown in Tables SI, S2, S3 and S4. We also give the official terms for each of the species, where applicable, from the following ontologies: UniProtKB/Swiss-Prot[3], Interpro[4], the Gene Ontology (GO)[5], and the Chemical Entities of Biological Interest (CHEBI) database[6]. A fully annotated model is available at the Biomodels database (ID:MODEL0912070000) [7,8].

**Rate laws, parameter values and sensitivity analysis**

As we used stochastic simulation, we used mass action kinetics for all the kinetic rate laws. Where possible, parameter values were taken from experimental data. There is data for the half-life of proteins and so we set protein degradation rates to match this. We tested the model output by setting the protein synthesis rate to zero and then checking the half-life of each protein and made adjustments where necessary. We then set the rates for protein synthesis and checked that protein levels remain constant over time under normal conditions, again making any necessary adjustments. All the reactions for protein degradation by the proteasome also contained an additional parameter, *k_proteff_*, which represents the efficiency of the proteasome and is initially set to one. We could then mimic proteasome inhibition, by reducing the value of *k_proteff_*. The parameters for aggregation were set to be very low as aggregation does not usually occur unless the concentration of the substrates is high. Therefore there is a long lag phase before any aggregates appear. Once an aggregate reaches a certain size, the process becomes much faster and depends on the level of SeqAggp (the species representing the inclusion body). ). The rate laws and parameter values are shown in Tables S1, S2, S3, S4, S5, S6 andS7.

The model contains a wide range of parameter rates which vary over ten orders of magnitude. This is because some reactions such as ubiquitination and proteasome activity occurring over timescales of seconds whereas aggregation occurs over time scales of days. Many of the species are also present at very low copy numbers. This means that the model is not suitable for deterministic simulation and so we use the Gillespie algorithm for the majority of the simulations. However, in order to carry out sensitivity analysis of all the parameter values it was not feasible to use stochastic simulation as the computing time to do this is prohibitive. So we used the parameter scan facility in COPASI 4.6 [9] which is based on a deterministic algorithm (LSODA). Each scan consisted of a time course calculation over 10 intervals of the chosen parameter ranging from half the initial parameter value to double the initial parameter value. The results were analysed for the level of SeqAggP at the time-point 30 hours and the difference between the level for the maximum value and minimum value of the the parameter was calculated. If the absolute difference in the level of SeqAggP was greater than 50, then we considered that the parameter had a large effect on inclusion formation. If the absolute difference was between 1 and 50, we considered the parameter to have a small effect on inclusion formation. If the absolute difference was less than one, then we considered that the parameter did not affect inclusion formation. The results are shown in Table S8, where the parameters are listed in the order of magnitude of their effect (largest effect at the top) and split into those that increased inclusion levels and those that decreased it. The parameter which has the largest positive effect on inclusion formation is *k_synNatP_*. This is because if the synthesis rate of protein is increased without also increasing its degradation rate, the pool of protein continues to increase with time and so there is much more protein which can be damaged and misfolded and the pool of misfolded protein also increases with time and then starts to aggregate. Once an aggregate forms, the misfolded protein is rapidly sequestered accounting for the much higher levels of SeqAggP. The parameter *k_growth1_* has a large effect as increasing the value of this parameter increases the rate at which misfolded protein is sequestered into inclusions and so more misfolded protein ends up in inclusions as opposed to being degraded or refolded. If *k_genROS_* is increased, then the level of ROS increases leading to more protein misfolding and so more aggregation. The effect of increasing *k_damUchl1_* is to increase the amount of damaged UCH-L1 which is also liable to aggregation. Damaged UCH-L1 also binds to Lamp2a receptors and inhibits degradation of α-synuclein which will then also be more liable to aggregate. Increasing the value of *k_synSUB_* has a similar effect to increasing *k_synNatP_* but the effect is much smaller as the pool of SUB (UCH-L1 substrate) is much smaller than the pool of NatP (the generic pool of protein). If the rate at which damaged UCH-L1 is released from Lamp2a receptors is increased (*k_relLamp2aUchl1dam_*), then more damaged UCH-L1 is available to form aggregates and not surprisingly increasing the binding rate (*k_binLamp2aUchl1dam_*) has the opposite effect. The parameter *k_agg1dam_* affects the rate of the first step in the aggregation process of damaged α-synuclein and damaged UCH-L1 and so we would expect that increasing this would lead to higher levels of inclusions. The parameter which has the largest effect in reducing inclusion levels is *k_binProt_*. This parameter affects the rate of proteins binding to the proteasome prior to degradation and so an increase in its level leads to more degradation and so reduces the pool of misfolded protein. Increasing the activity rate of the proteasome (*k_actProt_*) also leads to more protein degradation and smaller pools of misfolded protein. Increasing the rate of ROS removal leads to less protein damage occurring and so also reduces the pool of misfolded protein. The availability of ubiquitin has a small effect on inclusion formation with decreased pools of ubiquitin leading to more inclusions.

**Computer experiments to mimic UCH-L1 overexpression and proteasome inhibition**

In order to mimic proteasome inhibition (PI) at time, t = 20 hours, we used an SBML event structure with trigger t>=72000.0 and event assignment *k_proteff_*=0.3. This reduced the efficiency of all proteasome degradation reactions by 70%. To model the various levels of UCH-L1 in the cells as described in the main manuscript, we ran 50 simulations with basal levels of UCH-L1 and *k_synUCHL1_*=2.55E-2; 100 simulations with the initial amount of UCH-L1=12000 and *k_synUCHL1_*=5.1E-2; and 50 simulations with the initial amount of UCHL1=18000 and *k_synUCHL1_*=7.65E-2. We also varied these proportions to see how the model predictions were affected.

**Computer experiments to mimic UCH-L1 mutants and proteasome inhibition**

To mimic the experimental procedure, we assumed that the simulated cell contained endogenous UCH-L1 and was then transfected with the mutant form. As in the overexpression experiments, we assumed that 25% of cells were untransfected (i.e. had normal UCH-L1 levels); 50% fo the cells had equal proportions of wild-type and mutant forms of UCH-L1; and 25% of cells had twice as much mutant UCH-L1 as wild-type.

**I93M mutant – lower hydrolase activity**

We assumed that the I93M mutant has 55% lower hydrolase activity than wild-type and adjusted the parameter *k_actUchl1_* to account for the different proportions of wild-type and mutant UCH-L1 present.

**I93M mutant – lower hydrolase activity and behaves like oxidatively damaged UCH-L1**

We included an additional species UCHL1_mut to represent the I93M mutant and assumed that UCHL1_mut has a higher propensity to become damaged. We assumed that the turnover rates of UCHL1_mut are the same as the wild-type UCH-L1. The results are shown in Table S8 and Figure 6B of the main manuscript.

**S18Y mutant – higher hydrolase activity**

We assumed that the S18Y mutant has 20% higher hydrolase activity that wild-type and adjusted the parameter *k_actUchl1_* to account for the different proportions of wild-type and mutant UCH-L1 present. The results are shown in Table S9 and Figure 6B.

**Stochastic simulation**

We used stochastic simulation since random effects are important in this model and some of the species are present at low numbers of molecules, e.g. small aggregates. The parameters were based on experimental data and even under normal conditions, it is possible to get low levels of aggregated protein. We ran 100 simulations for the model under normal conditions and plotted the mean value of some of the species (Figure S1). It can be seen from panel C that a small percentage of cells end up with one or two aggregated proteins. If the model is run with a deterministic simulator, the system will not reach a steady state due to very low levels of aggregated protein that start to form. Therefore we recommend that this model is only run with a stochastic simulator. We used Gillespie’s direct method and brief description of the algorithm is given below.

Gillespie algorithm:

0) **Initialization**: Initialize the number of molecules in the system, parameter values, and random number generators.

1) **Monte Carlo Step**: Generate random numbers to determine the next reaction to occur as well as the time interval.

2) **Update**: Increase the time step by the randomly generated time in Step 1. Update the molecule count based on the reaction that occurred.

3) **Iterate**: Go back to Step 1 unless the number of reactants is zero or the simulation time has been exceeded.

**References**
