# Supplementary figures and images for: Modelling the Role of UCH-L1 on Protein Aggregation in Age-Related Neurodegeneration
Source: PLoS One. 2010 Oct 6;5(10):e13175. doi: 10.1371/journal.pone.0013175 (PMC2950841; doi:10.1371/journal.pone.0013175)

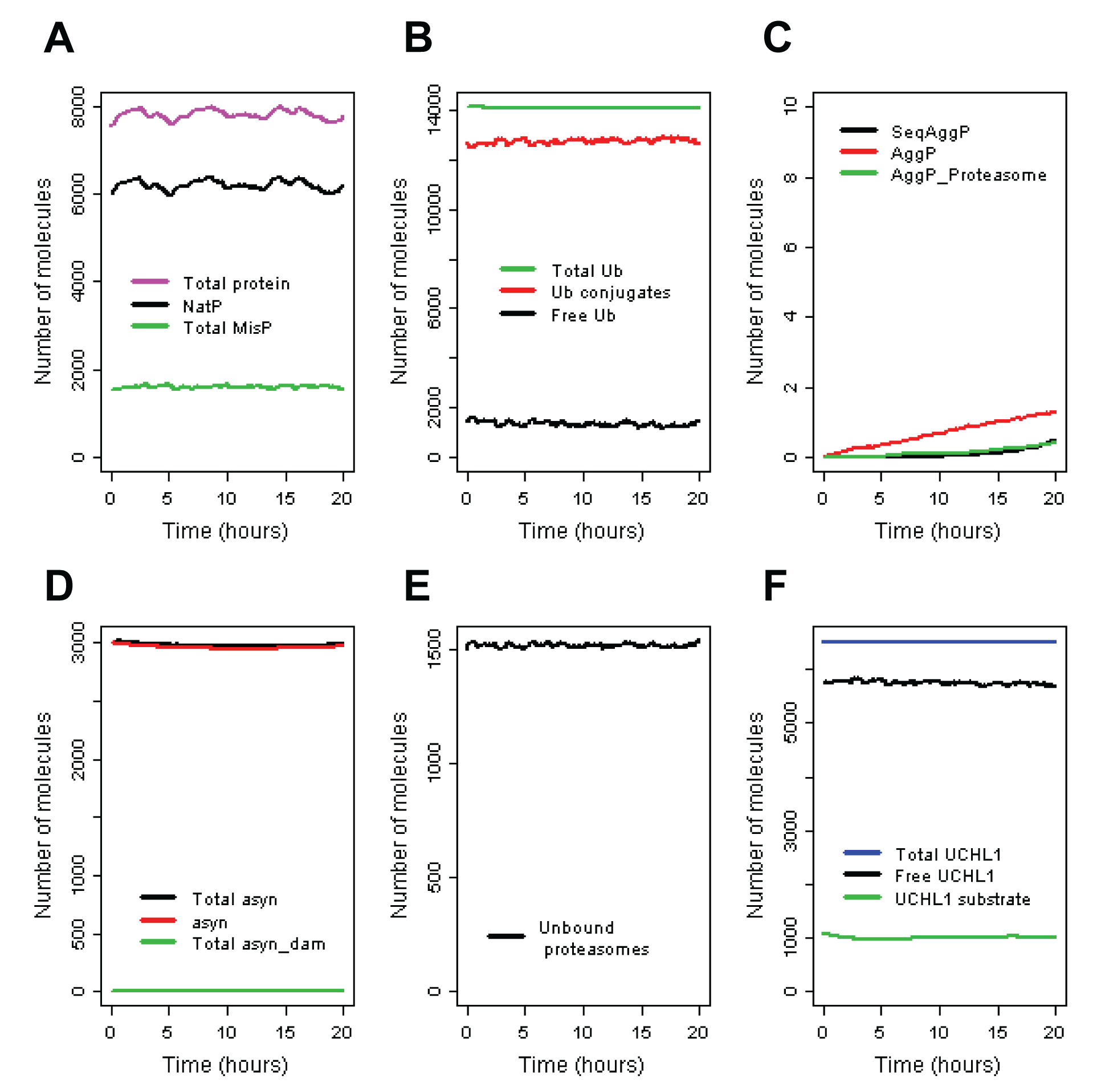

Supplement: Figure S1 — Plot of mean values for 100 runs of model under normal conditions. A Generic pool of protein. NatP = Native protein; TotalMisP = unbound misfolded protein + all bound forms of misfolded protein; Total protein = NatP + TotalMisP. B Ubiquitin pools. Ub conjugates includes all complexes containing ubiquitin not just ubiquitinated proteins (e.g. Ub-UCHL1 complex is included in this pool). C Aggregated protein. SeqAggP = aggregates sequestered into inclusion bodies; AggP = small unbound aggregates (of all types); AggP_Proteasome = small aggregates bound to the proteasome. D α-synuclein levels: asyn = unbound α-synuclein; asyn_dam = total pool of damaged α-synuclein (except any that is present in inclusions); total asyn = total pool of α-synuclein. E Pool of unbound proteasomes. F UCHL1 pools: Free UCHL1 = unbound UCHL1; Total UCHL1 = total pool of UCHL1 (except any that is present in inclusions); UCHL1 substrate = total level of the UCHL1 substrate (either bound or unbound) (0.17 MB TIF) [file pone.0013175.s002.tif]
