# Supplementary material for: Modelling the Role of UCH-L1 on Protein Aggregation in Age-Related Neurodegeneration
Source: PLoS One. 2010 Oct 6;5(10):e13175. doi: 10.1371/journal.pone.0013175 (PMC2950841; doi:10.1371/journal.pone.0013175)
Supplement: Table S1 — Model species for generic UPS component. (0.05 MB DOC) [file pone.0013175.s003.doc]

**Table S1 Model species for generic UPS component**

| Species description | Species Name | Database term | Initial Amount^a^ |
| --- | --- | --- | --- |
| Native protein | NatP | N/A | 6000 |
| Misfolded protein | MisP | N/A | 80 |
| Ubiquitin | Ub | P62988 | 1500 |
| Ubiquitin activating enzyme | E1 | IPR000011 | 105 |
| Ubiquitin conjugating enzyme | E2 | IPR000608 | 50 |
| Ubiquitin ligase (HECT) | E3 | IPR000569 | 300 |
| De-ubiquitinating enzyme | DUB | IPR001394 | 160 |
| Proteasome | Proteasome | GO:0000502 | 1500 |
| Reactive oxygen species | ROS | CHEBI:26523 | 10 |
| Adenosine triphosphate | ATP | CHEBI:15422 | 10000 |
| Adenosine diphosphate | ADP | CHEBI:16761 | 1000 |
| Adenosine monophosphate | AMP | CHEBI:22254 | 1000 |
| Misfolded protein bound by E3 | E3_MisP | IPR000569 | 2 |
| E1 bound by Ub | E1_Ub | IPR000011, P62988 | 795 |
| E2 bound by Ub | E2_Ub | IPR000608, P62988 | 950 |
| Monoubiquitinated misfolded protein | E3_MisP_Ub | IPR000569, P62988 | 0 |
| Polyubiquitinated misfolded protein | E3_MisP_Ub2, … E3_MisP_Ub8 | IPR000569, P62988 | 0,0,0,0,0,0,300 |
| Mono- and Poly-ubiquitinated misfolded protein bound by DUB | E3_MisP_Ub_DUB, … E3_MisP_Ub8_DUB | IPR000569, P62988, IPR001394 | 100 |
| Polyubiquitinated protein bound to proteasome | MisP_Ub4_Proteasome, …  MisP_Ub8_Proteasome | P62988  GO:0000502 | 0,1,2,10, 350 |
| Aggregated protein | AggP1, …, AggP5 | N/A | 0 |
| Sequestered aggregated protein | SeqAggP | N/A | 0 |
| Aggregated protein bound to proteasome | AggP_Proteasome | GO:0000502 | 0 |

^a^Units: number of molecules

N/A not applicable

IPR: InterPro (<http://www.ebi.ac.uk/interpro/>)

GO: Gene ontology ([www.geneontology.org](http://www.geneontology.org/))

CHEBI: Chemical Entities of Biological Interest database ([www.ebi.ac.uk/chebi](http://www.ebi.ac.uk/chebi) )

P terms: UniProtKB/Swiss-Prot (<http://www.uniprot.org/>)
