# Supplementary material for: Modelling the Role of UCH-L1 on Protein Aggregation in Age-Related Neurodegeneration
Source: PLoS One. 2010 Oct 6;5(10):e13175. doi: 10.1371/journal.pone.0013175 (PMC2950841; doi:10.1371/journal.pone.0013175)
Supplement: Table S2 — Model species for UCHL1 turnover, activity, damage and aggregation. (0.04 MB DOC) [file pone.0013175.s004.doc]

**Table S2 Model species for UCHL1 turnover**, activity, damage and aggregation

| Species description | Species Name | Database term | Initial Amount |
| --- | --- | --- | --- |
| UCH-L1 | UCHL1 | P09936 | 5785 |
| UCH-L1 bound to ubiquitin | Ub_UCHL1 | P62988, P09936 | 500 |
| UCH-L1 bound to proteasome | UCHL1_Proteasome | P09936, GO:0000502 | 10 |
| Damaged UCH-L1 | UCHL1_damaged | P09936 | 0 |
| Damaged UCH-L1 bound to proteasome | UCHL1_damaged_Proteasome | P09936, GO:0000502 | 0 |
| Lysosome | Lysosome | GO:0005764 | 1200 |
| Lamp2a receptor | Lamp2a | P13473 | 200 |
| Damaged UCH-L1 bound to Lamp2a receptor | Lamp2a_UCHL1_damaged | P13473, P09936 | 0 |
| UCH-L1 substrate | SUB | N/A | 815 |
| UCH-L1 substrate in misfolded state | SUB_misfolded | N/A | 5 |
| E3 ligase for UCH-L1 substrate | E3SUB | IPR000569 | 160 |
| E3 ligase bound to misfolded substrate | E3SUB_SUB_misfolded | IPR000569 | 5 |
| Ubiquitinated substrate | E3SUB_SUB_misfolded_Ub | IPR000569, P62988 | 0 |
| Polyubiquitinated substrate | E3SUB_SUB_misfolded_Ub(X) (X=2-8) | IPR000569, P62988 | 0,0,0,0,0,0,15 |
| UCH-L1 bound to ubiquitinated substrate | E3SUB_SUB_misfolded_Ub_UCHL1 | IPR000569, P62988, P09936 | 20 |
| UCH-L1 bound to polyubiquitinated substrate | E3SUB_SUB_misfolded_Ub(X)_UCHL1  (X=2-8) | IPR000569, P62988, P09936 | 20,25,25,30, 40,40 |
| Polyubiquitinated substrate bound to proteasome | SUB_misfolded_Ub(X)_Proteasome  (X=4-8) | P62988, GO:0000502 | 0,0,0,5,20 |
| Small aggregate of damaged UCH-L1 | AggU1, …, AggU5 | P09936 | 0 |
| Small aggregate of misfolded UCH-L1 substrate | AggS1, …, AggS1 | N/A | 0 |

IPR: InterPro (<http://www.ebi.ac.uk/interpro/>)

GO: Gene ontology ([www.geneontology.org](http://www.geneontology.org/))

P terms: UniProtKB/Swiss-Prot (<http://www.uniprot.org/>)
