# Supplementary material for: Modelling the Role of UCH-L1 on Protein Aggregation in Age-Related Neurodegeneration
Source: PLoS One. 2010 Oct 6;5(10):e13175. doi: 10.1371/journal.pone.0013175 (PMC2950841; doi:10.1371/journal.pone.0013175)
Supplement: Table S3 — Model species for α-synuclein turnover, damage and aggregation. (0.04 MB DOC) [file pone.0013175.s005.doc]

**Table S3 Model species for α-synuclein turnover, damage and aggregation**

| Species description | Species Name | Database term | Initial Amount |
| --- | --- | --- | --- |
| α-synuclein | asyn | P37840 | 3000 |
| α-synuclein bound to proteasome | asyn_Proteasome | P37840, GO:0000502 | 0 |
| α-synuclein bound to Lamp2a receptor | asyn_Lamp2a | P37840, P13473 | 0 |
| Damaged α-synuclein | asyn_dam | P37840 | 0 |
| E3 ligase, Parkin | Parkin | O60260 | 4800 |
| Parkin bound to damaged α-synuclein | Parkin_asyn_dam | O60260, P37840 | 0 |
| Ubiquitinated damaged α-synuclein | Parkin_asyn_dam_Ub | O60260, P37840, P62988 | 0 |
| Polyubiquitinated damaged α-synuclein | Parkin_asyn_dam_Ub(X) (X=2-8) | O60260, P37840, P62988 | 0 |
| DUB bound to ubiquitinated damaged  α-synuclein | Parkin_asyn_dam_Ub_DUB | O60260, P37840, P62988, IPR001394 | 0 |
| DUB bound to polyubiquitinated damaged  α-synuclein | Parkin_asyn_dam_Ub(X)_DUB (X=2-8) | O60260, P37840, P62988, IPR001394 | 0 |
| Polyubiquitinated damaged α-synuclein bound to proteasome | asyn_dam_Ub(X)_Proteasome (X=4-8) | P37840, P62988, GO:0000502 | 0 |
| Small aggregate of α-synuclein | AggA1, …, AggA5 | P37840 | 0 |
| Small aggregate of damaged α-synuclein | AggD1, …, AggD5 | P37840 | 0 |

IPR: InterPro (<http://www.ebi.ac.uk/interpro/>)

GO: Gene ontology ([www.geneontology.org](http://www.geneontology.org/))

O, P terms: UniProtKB/Swiss-Prot (<http://www.uniprot.org/>)
