# Supplementary material for: Modelling the Role of UCH-L1 on Protein Aggregation in Age-Related Neurodegeneration
Source: PLoS One. 2010 Oct 6;5(10):e13175. doi: 10.1371/journal.pone.0013175 (PMC2950841; doi:10.1371/journal.pone.0013175)
Supplement: Table S4 — Dummy species used to identify different proteins in inclusions. (0.03 MB DOC) [file pone.0013175.s006.doc]

**Table S4 Dummy species used to identify different proteins in inclusions**

| Species description | Species Name | Initial Amount |
| --- | --- | --- |
| α-synuclein | aggasyn | 0 |
| Damaged α-synuclein | aggasyndam | 0 |
| Parkin | aggParkin | 0 |
| Ubiquitin | aggUb | 0 |
| E3 ligase | aggE3 | 0 |
| DUB | aggDUB | 0 |
| Generic misfolded protein | aggMisP | 0 |
| UCH-L1 | aggUchl1 | 0 |
| Damaged UCH-L1 | aggUchl1dam | 0 |
| UCH-L1 substrate | aggSUB | 0 |
