# Supplementary material for: Modelling the Role of UCH-L1 on Protein Aggregation in Age-Related Neurodegeneration
Source: PLoS One. 2010 Oct 6;5(10):e13175. doi: 10.1371/journal.pone.0013175 (PMC2950841; doi:10.1371/journal.pone.0013175)
Supplement: Table S5 — Reactions for generic UPS component. (0.07 MB DOC) [file pone.0013175.s007.doc]

**Table S5 List of reactions for generic UPS component**

| Reaction | Reactants and Products | Kinetic rate law | Value^a^ |
| --- | --- | --- | --- |
| Protein synthesis | Source→ NatP | *k_synNatP_* | 2.4molecule.s^-1^ |
| Misfolding | NatP+ROS→ MisP+ROS | *k_misfold_*<#NatP><#ROS> | 4.0E-5molecule^-1^s^-1^ |
| Refolding | MisP→ NatP | *k_refold_*<#MisP> | 8.0E-5s^-1^ |
| MisP/E3 binding | E3+MisP→ E3_MisP | *k_binMisPE3_*<#E3><#MisP> | 1.0E-4molecule^-1^s^-1^ |
| MisPE3 release | E3_MisP→ E3+MisP | *k_rel MisPE3_*<#E3_MisP> | 2.0E-4s^-1^ |
| E1/Ub binding | E1+Ub+ATP→ E1_Ub+AMP | *k_binE1Ub_*<#E1><#Ub> <#ATP>/(5000+<#ATP>) | 2.0E-4molecule^-1^s^-1^ |
| E2/Ub binding | E2+E1_Ub→ E1+E2_Ub | *k_binE2Ub_*<#E2><#E1_Ub> | 1.0E-3molecule^-1^s^-1^ |
| Monoubiquitination | E2_Ub+E3_MisP→ E2+E3_MisP_Ub | *k_monoUb_*<#E2_Ub><#E3_MisP> | 1.0E-3molecule^-1^s^-1^ |
| Polyubiquitination1 | E2_Ub+E3_MisP_Ub→E2+ E3_MisP_Ub2 | *k_polyUb_*<#E2_Ub><#E3_MisP_Ub> | 1.0E-2molecule^-1^s^-1^ |
| PolyubiquitinationX (X=2-7) | E2_Ub+E3_MisP_Ub(X)→E2+ E3_MisP_Ub(X+1) | *k_polyUb_* <#E2_Ub><#E3_MisP_Ub(X)> | 1.0E-2molecule^-1^s^-1^ |
| MisP DUB bindingX (X=1-8) | DUB+E3_ MisP_Ub(X)→ E3_MisP_Ub(X)_DUB | *k_binMisPDUB_*<#DUB><#E3_MisP_Ub8> | 2.0E-7molecule^-1^s^-1^ |
| De-ubiquitinationX (X=2-8) | E3_ MisP_Ub(X)_DUB→ E3_ MisP_Ub(X-1)_DUB+Ub | *k_actDUB_*<#E3_MisP_Ub(X)_DUB> | 1.0E-4s^-1^ |
| De-ubiquitination1 | E3_MisP_Ub_DUB→ DUB+E3_MisP+Ub | *k_actDUB_*<#E3_MisP_Ub_DUB> | 1.0E-4s^-1^ |
| Proteasome bindingX (X=4-8) | E3_MisP_Ub(X)+Proteasome→ MisP_Ub(X)_ Proteasome+E3 | *k_binProt_*<#E3_MisP_Ub(X)><#Proteasome> | 5.0E-6molecule^-1^s^-1^ |
| De-ubiquitination-BoundMisPUb4 | DUB+ MisP_Ub4_Proteasome→ DUB+ MisP+4Ub+ Proteasome | *k_actDUBProt_*<#DUB><#MisP_Ub4_Proteasome> | 1.0E-6molecule^-1^s^-1^ |
| De-ubiquitination-BoundMisPUbX  (X=5-8) | DUB+ MisP_Ub(X)_Proteasome→ DUB+MisP_Ub(X-1)_Proteasome+Ub | *k_actDUBProt_*<#DUB><#MisP_Ub(X)_Proteasome> | 1.0E-6molecule^-1^s^-1^ |
| ProteasomeActivityX (X=4-8) | ATP+MisP_Ub(X)_ Proteasome→ ADP+Proteasome+(X)Ub, | *k_actProt_ *k_proteff_* <#MisP_Ub(X)_Proteasome> <#ATP>/(5000+<#ATP>) | 1.0E-2s^-1^, 1.0 |
| Aggregation1 | 2MisP→AggP1 | *k_agg1_*<#MisP><#MisP-1>/2.0 | 1.0E-12molecule^-1^s^-1^ |
| Aggregation(X+1) (X=1-4) | MisP+AggP(X)→AggP(X+1) | *k_agg2_*<#MisP><#AggP(X)> | 1.0E-10molecule^-1^s^-1^ |
| Disaggregation1 | AggP1→2MisP | *k_disagg1_*<#AggP1> | 1.0E-8s^-1^ |
| Disaggregation2 | AggP2→AggP1+MisP | *k_disagg2_*<#AggP2> | 8.0E-9s^-1^ |
| Disaggregation3 | AggP3→AggP2+MisP | *k_disagg3_*<#AggP3> | 6.0E-9s^-1^ |
| Disaggregation4 | AggP4→AggP3+MisP | *k_disagg4_*<#AggP4> | 4.0E-9s^-1^ |
| Disaggregation5 | AggP5→AggP4+MisP | *k_disagg5_*<#AggP5> | 2.0E-9s^-1^ |
| Inclusion formation | AggP5+MisP→SeqAggP^a^ | *k_agg2_*<#MisP><#AggP5> | 1.0E-10molecule^-1^s^-1^ |
| Inclusion growth1 | SeqAggP+MisP→2SeqAggP^a^ | *k_igrowth1_*<#MisP><#SeqAggP> | 5.0E-9molecule^-1^s^-1^ |
| Inclusion growth2 | SeqAggP+E3_MisP→2SeqAggP^a^ | *k_igrowth2_*<#E3_MisP><#SeqAggP> | 5.0E-9molecule^-1^s^-1^ |
| Inclusion growth(X+2) (X=1-8) | SeqAggP+E3_MisP_Ub(X)→2SeqAggP^a^ | *k_igrowth2_*<#E3_MisP_Ub(X)><#SeqAggP> | 5.0E-9molecule^-1^s^-1^ |
| Inclusion growth(X+10) (X=1-8) | SeqAggP+E3_MisP_Ub(X)_DUB→2SeqAggP^a^ | *k_igrowth2_*<#E3_MisP_Ub(X)_DUB><#SeqAggP> | 5.0E-9molecule^-1^s^-1^ |
| Proteasome inhibition(X) (X=1-5) | AggP(X)+Proteasome→AggP_Proteasome | *k_binAggProt_*<#AggP(X)><#Proteasome> | 5.0E-9molecule^-1^s^-1^ |
| ROS generation Agg(X) (X=1-5) | AggP(X) →AggP(X)+ROS | *k_genROSAggP_*<#AggP(X)> | 2.0E-5s^-1^ |
| ROS generation | Source→ROS | *k_genROS_* | 1.0E-2molecule.s^-1^ |
| ROS removal | ROS→Sink | *k_remROS_*<#ROS> | 1.0E-3s^-1^ |
| Ubiquitin synthesis | Source→Ub | *k_ubs_* | 1.2E-2molecule.s^-1^ |
| Ubiquitin degradation | Ub+Proteasome→Proteasome | *k_ubd_*k_proteff_* <#Proteasome><#Ub> | 4.4E-9 molecule^-1^s^-1^, 1.0 |
| Ubiquitin upregulation | MisP→MisP+3Ub | *k_ubss_*<#MisP^6>/(1500^6+<#MisP^6>) | 0.1molecules^-1^ |

^a^Note that products also include dummy species to keep track of which proteins end up in inclusions
