# Supplementary material for: Modelling the Role of UCH-L1 on Protein Aggregation in Age-Related Neurodegeneration
Source: PLoS One. 2010 Oct 6;5(10):e13175. doi: 10.1371/journal.pone.0013175 (PMC2950841; doi:10.1371/journal.pone.0013175)
Supplement: Table S6 — Reactions for UCH-L1 turnover, activity, damage and aggregation. (0.06 MB DOC) [file pone.0013175.s008.doc]

**Table S6** Reactions for UCH-L1 turnover, activity, damage and aggregation

| Reaction | Reactants and Products | Kinetic rate law | Value^a^ |
| --- | --- | --- | --- |
| UCH-L1 synthesis | Source→UCHL1 | *k_synUCHL1_* | 2.2E-2molecule.s^-1^ |
| UCH-L1 binding to proteasome | UCHL1+Proteasome→UCHL1_Proteasome | *k_binUCHL1Prot_*<#UCHL1><#Proteasome> | 4.0E-10molecule^-1^s^-1^ |
| UCH-L1 proteasomal degradation | UCHL1_Proteasome → Proteasome | *k_degProtUCHL1_*k_proteff_* <#UCHL1_Proteasome> | 1.0E-2s^-1^, 1.0 |
| UCH-L1 lysosomal degradation | UCHL1+Lysosome→Lysosome | *k_degLysUCHL1_*<#UCHL1><#Lysosome> | 2.7E-9molecule^-1^s^-1^ |
| UCH-L1 damage | UCHL1+ROS→UCHL1_dam+ROS | *k_damUCHL1_*<#UCHL1><#ROS> | 1.0E-8molecule^-1^s^-1^ |
| Damaged UCH-L1 binding to proteasome | UCHL1_dam+Proteasome→  UCHL1_dam_Proteasome | *k_binUCHL1Prot_*<#UCHL1_dam><#Proteasome> | 4.0E-10molecule^-1^s^-1^ |
| Damaged UCH-L1 proteasomal degradation | UCHL1_dam_Proteasome →Proteasome | *k_degProtUCHL1_*k_proteff_* <#UCHL1_dam_Proteasome> | 1.0E-2s^-1^, 1.0 |
| Damaged UCH-L1 lysosomal degradation | UCHL1_dam+Lysosome →Lysosome | *k_degLysUCHL1dam_*<#UCHL1_dam><#Lysosome> | 2.7E-9molecule^-1^s^-1^ |
| UCH-L1 binding to Lamp2a | UCHL1_dam+Lamp2a→ Lamp2a_UCHL1_dam | *k_binLamp2aUCHL1dam_*<#UCHL1_dam><#Lamp2a> | 1.0E-5molecule^-1^s^-1^ |
| UCH-L1-Lamp2a release | Lamp2a_UCHL1_dam→ UCHL1_dam+Lamp2a | *k_relLamp2aUCHL1dam_*<#Lamp2a_UCHL1_dam> | 5.0E-5s^-1^ |
| Ub-UCH-L1 binding | Ub+UCHL1→ Ub_UCHL1 | *k_binUbUCHL1_*<#Ub><#UCHL1> | 3.0E-6molecule^-1^s^-1^ |
| Ub-UCH-L1 release | Ub_UCHL1→ Ub+UCHL1 | *k_relUbUCHL1_*<#Ub_UCHL1> | 5.0E-2s^-1^ |
| Damaged UCH-L1 aggregation1 | 2 UCHL1_dam→AggU1 | *k_agg1dam_*<#UCHL1_dam><# UCHL1_dam -1>/2.0 |  |
| Damaged UCH-L1 aggregationX (X=2-5)^b^ | UCHL1_dam+AggU(X-1) →AggU(X) | *k_agg2dam_*<#UCHL1_dam><#AggU(X)> |  |
| UCH-L1 substrate synthesis | Source→SUB | *k_synSUB_* | 1.3E-1molecule.s^-1^ |
| SUB misfolding | SUB+ROS→SUB_misfolded+ROS | *k_misfoldSUB_*<#SUB><#ROS> | 2.0E-5molecule^-1^s^-1^ |
| SUB refolding | SUB_misfolded→SUB | *k_refoldSUB_*<#SUB_misfolded> | 5.0E-5s^-1^ |
| SUB-E3SUB binding | SUB_misfolded+E3SUB→E3SUB_SUB_misfolded | *k_binE3SUB_*<#E3SUB><#SUB_misfolded> | 5.0E-4molecule^-1^s^-1^ |
| SUB-E3SUB release | E3SUB_SUB_misfolded → SUB_misfolded +E3SUB | *k_relE3SUB_*<#E3SUB_SUB_misfolded > | 2.0E-4s^-1^ |
| SUB ubiquitination^b^ | E3SUB_SUB_misfolded+E2_Ub→ E3SUB_SUB_misfolded_Ub+E2 | *k_monoUb_*<#E3SUB_SUB_misfolded > <#E2_Ub> | 1.0E-3molecule^-1^s^-1^ |
| SUB – UCHL1 bindingX (X=1-8) | E3SUB_SUB_misfolded_Ub(X)+UCHL1→ E3SUB_SUB_misfolded_Ub(X)_UCHL1 | *k_binSUBUCHL1_*<#E3SUB_SUB_misfolded_Ub(X)> <#UCHL1> | 4.6E-8molecule^-1^s^-1^ |
| SUB de-ubiquitinationX (X=1-8) | E3SUB_SUB_misfolded_Ub(X)_UCHL1→ E3SUB_SUB_misfolded_Ub(X-1)_UCHL1+Ub | *k_actUchl1_*<#E3SUB_SUB_misfolded_Ub(X)_UCHL1> | 1.0E-4s^-1^ |
| SUB – proteasome bindingX (X=4-8) | E3SUB_SUB_misfolded_Ub(X)+Proteasome→ SUB_misfolded_Ub(X)_Proteasome+E3SUB | *k_binProt_*<#E3SUB_SUB_misfolded_Ub(X)> <#Proteasome> | 5.0E-6molecule^-1^s^-1^ |
| De-ubiquitination bound SUB4 | SUB_misfolded_Ub4_Proteasome+DUB→  SUB_misfolded+Proteasome+DUB+4Ub | *k_actDUBProt_*<#SUB_misfolded_Ub4_Proteasome> <#DUB> | 1.0E-6molecule^-1^s^-1^ |
| De-ubiquitination bound SUBX (X=5-8) | SUB_misfolded_Ub(X)_Proteasome+DUB→  SUB_misfolded_Ub(X-1)_Proteasome+DUB+Ub | *k_actDUBProt_*<#SUB_misfolded_Ub(X)_Proteasome> <#DUB> | 1.0E-6molecule^-1^s^-1^ |
| SUB degradationX (X=4-8) | SUB_misfolded_Ub(X)_Proteasome+ATP→  (X)Ub+Proteasome+ADP | *k_actProt_*k_proteff_* <#SUB_misfolded_Ub(X)_Proteasome> <#ATP>/(5000+<#ATP>) | 1.0E-2s^-1^, 1.0 |
| SUB aggregation1 | 2SUB_misfolded→AggS1 | *k_aggSUB1_*<#SUB_misfolded><#SUB_misfolded-1>/2.0 | 1.0E-12molecule^-1^s^-1^ |
| SUB aggregationX (X=2-5)^b^ | SUB_misfolded+AggS(X-1)→AggS(X) | *k_aggSUB2_*<#SUB_misfolded><#AggS(X)> | 1.0E-10molecule^-1^s^-1^ |

^a^only 1^st^ step is shown as steps are identical as for MisP in Table S5. ^b^Reactions for UCH-L1_dam and SUB disaggregation, inclusion formation and growth, proteasome inhibition and ROS production are similar to those for MisP in Table S5.
