# Supplementary material for: Modelling the Role of UCH-L1 on Protein Aggregation in Age-Related Neurodegeneration
Source: PLoS One. 2010 Oct 6;5(10):e13175. doi: 10.1371/journal.pone.0013175 (PMC2950841; doi:10.1371/journal.pone.0013175)
Supplement: Table S7 — Reactions for α-synuclein turnover, damage and aggregation. (0.06 MB DOC) [file pone.0013175.s009.doc]

**Table S7 Reactions for α**-synuclein turnover, damage and aggregation

| Reaction | Reactants and Products | Kinetic rate law | Value^a^ |
| --- | --- | --- | --- |
| synthesis | Source→asyn | *k_synasyn_* | 2.8E-2molecule.s^-1^ |
| 20S proteasome binding | asyn+Proteasome→asyn_Proteasome | *k_binasynProt_*<#asyn><#Proteasome> | 1.7E-9molecule^-1^s^-1^ |
| 20S proteasome degradation | asyn_Proteasome→Proteasome | *k_degasynProt_*k_proteff_* <#asyn_Proteasome> | 1.0E-2s^-1^, 1.0 |
| Lamp2a binding | asyn+Lamp2a→asyn_Lamp2a | *k_binasynLamp2a_*<#asyn><#Lamp2a> | 4.0E-8s^-1^ |
| CMA degradation | asyn_Lamp2a → Lamp2a | *k_CMAasyn_*<#asyn_Lamp2a> | 1.0E-3s^-1^ |
| Damage | asyn+ROS→asyn_dam+ROS | *k_damasyn_*<#asyn><#ROS> | 5.0E-8molecule^-1^s^-1^ |
| Parkin binding | asyn_dam+Parkin→ Parkin_asyn_dam | *k_binasynParkin_*<#asyn_dam><#Parkin> | 1.0E-4molecule^-1^s^-1^ |
| Parkin release | Parkin_asyn_dam → asyn_dam+Parkin | *k_relasynParkin_*<#Parkin_asyn_dam> | 2.0E-4s^-1^ |
| Monoubiquitination | Parkin_asyn_dam +E2_Ub→ Parkin_asyn_dam_Ub+E2 | *k_monoUb_*<# Parkin_asyn_dam ><#E2_Ub> | 1.0E-3molecule^-1^s^-1^ |
| PolyubiquitinationX (X=1-7) | Parkin_asyn_dam _Ub(X)+E2_Ub→ Parkin_asyn_dam_Ub(X+1)+E2 | *k_polyUb_*<# Parkin_asyn_dam _Ub(X)><#E2_Ub> | 1.0E-2molecule^-1^s^-1^ |
| DUB bindingX (X=1-8) | Parkin_asyn_dam _Ub(X)+DUB→ Parkin_asyn_dam_Ub(X)_DUB | *k_binasynDUB_*<# Parkin_asyn_dam _Ub(X)><#DUB> | 2.0E-7molecule^-1^s^-1^ |
| De-ubiquitinationX (X=1-8) | Parkin_asyn_dam_Ub(X)_DUB →  Parkin_asyn_dam_Ub(X-1)_DUB+Ub | *k_actDUB_*<# Parkin_asyn_dam _Ub(X)_DUB> | 1.0E-4s^-1^ |
| Proteasome bindingX (X=4-8) | Parkin_asyn_dam _Ub(X)+Proteasome→  asyn_dam _Ub(X)_Proteasome+Parkin | *k_binProt_*<# Parkin_asyn_dam _Ub(X)><#Proteasome> | 5.0E-6molecule^-1^s^-1^ |
| De-ubiquitination Bound asyn4 | asyn_dam _Ub4_Proteasome +DUB→  asyn_dam+Proteasome+DUB+4Ub | *k_actDUBProt_*<#asyn_dam _Ub4_Proteasome><#DUB> | 1.0E-6molecule^-1^s^-1^ |
| De-ubiquitination Bound asynX (X=5-8) | asyn_dam _Ub(X)_Proteasome +DUB→  asyn_dam _Ub(X-1)_Proteasome+DUB+Ub | *k_actDUBProt_*<#asyn_dam _Ub(X)_Proteasome><#DUB> | 1.0E-6molecule^-1^s^-1^ |
| 26S proteasome degradationX (X=4-8) | asyn_dam _Ub(X)_Proteasome+ATP → Proteasome+(X)Ub+ADP | *k_actProt_*k_proteff_* <#asyn_dam _Ub(X)_Proteasome> <#ATP>/(5000+<#ATP>) | 1.0E-2s^-1^, 1.0 |
| Aggregation asyn1^a^ | 2asyn→AggA1 | *k_aggasyn1_*<#asyn><#asyn-1>/2.0 | 5.0E-12molecule^-1^s^-1^ |
| Aggregation asynX (X=2-5) ^a^ | asyn+AggA(X-1)→AggA(X) | *k_aggasyn2_*<#asyn><#AggA(X-1)> | 5.0E-10molecule^-1^s^-1^ |
| Disaggregation asyn1 ^a^ | AggA1→2asyn | *k_disaggasyn1_*<#AggA1> | 1.0E-8s^-1^ |
| Disaggregation asynX (X=2-5) ^a^ | AggA(X)→AggA(X-1)+asyn | *k_disaggasynX_*<#AggA(X)> | as in Table S5 |
| ProteasomeInhibition AggAX (X=1-5) ^a^ | AggA(X)+Proteasome→AggP_Proteasome | *k_binAggProt_*<#AggA(X)><#Proteasome> | 5.0E-9molecule^-1^s^-1^ |
| Inclusion formation ^a^ | asyn+AggA5→SeqAggP | *k_aggasyn2_*<#asyn><#AggA5> | 5.0E-10molecule^-1^s^-1^ |
| Inclusion growth ^a^ | asyn+SeqAgg→2SeqAggP | *k_igrowth1_*<#asyn><#SeqAggP> | 5.0E-9molecule^-1^s^-1^ |
| ROS generationX (X=1-5) ^a^ | AggA(X) →AggA(X)+ROS | *k_genROSAggP_*<#AggA(X)> | 2.0E-5s^-1^ |

^a^mol=molecules ^b^There is a identical set of reactions for damaged α-synuclein (asyn_dam) by replacing asyn with asyn_dam and AggA(X) with AggD(X). Reaction rates are kaggdam1=1.0E-5 and kaggdam2=5.0E-5, other reactions rates are the same as for undamaged α-synuclein. In addition asyn_dam bound to Parkin and ubiquitinated asyn-dam may be sequestered into inclusions.
