# Supplementary material for: Modelling the Role of UCH-L1 on Protein Aggregation in Age-Related Neurodegeneration
Source: PLoS One. 2010 Oct 6;5(10):e13175. doi: 10.1371/journal.pone.0013175 (PMC2950841; doi:10.1371/journal.pone.0013175)
Supplement: Table S8 — List of parameters which have an effect on inclusion formation. (0.03 MB DOC) [file pone.0013175.s010.doc]

**Table S8 Sensitivity Analysis**

| **Parameters which have large positive effect on inclusion formation** | **Parameters which have large negative effect on inclusion formation** | **Parameters which have small positive effect on inclusion formation** | **Parameters which have small negative effect on inclusion formation** |
| --- | --- | --- | --- |
| *k_synNatP_* | *k_binProt_* | *k_binSUBUchl1_* | *k_actDUB_* |
| *k_growth1_* | *k_remROS_* | *k_synUchl1_* | *k_degLysUchl1_* |
| *k_genROS_* | *k_actProt_* | *k_synasyn_* | *k_CMAasyn_* |
| *k_damUchl1_* | *k_binLamp2aUchl1dam_* | *k_growth2_* | *k_ubss_* |
| *k_synSUB_* |  | *k_binMisPDUB_* | *k_ubs_* |
| *k_relLamp2aUchl1dam_* |  | *k_misfoldSUB_* | *k_refoldSUB_* |
| *k_agg1dam_* |  | *k_relUbUchl1_* | *k_binUbUchl1_* |
|  |  | *k_misfold_* | *k_binasynProt_* |
|  |  | *k_ubd_* | *k_refold_* |
|  |  |  | *k_binUchl1Prot_* |
