# Supplementary material for: Modelling the Role of UCH-L1 on Protein Aggregation in Age-Related Neurodegeneration
Source: PLoS One. 2010 Oct 6;5(10):e13175. doi: 10.1371/journal.pone.0013175 (PMC2950841; doi:10.1371/journal.pone.0013175)
Supplement: Table S9 — Results for I93M mutant. Mutant has lower hydrolase activity and has 50% higher damage rate than wild-type. (0.03 MB DOC) [file pone.0013175.s011.doc]

**Table S9 Results for I93M mutant. Mutant has lower hydrolase activity and has 50% higher damage rate than wild-type**

|  | Number of “simulated cells” with inclusions | | | |
| --- | --- | --- | --- | --- |
| UCH-L1 expression  (Number of runs) | 4h post PI | 6h post PI | 8h post PI | No PI |
| 3x baseline (25) | 15 | 24 | 25 | 24 |
| 2x baseline (50) | 3 | 14 | 34 | 7 |
| Baseline (25) | 0 | 0 | 3 | 0 |
| Total (100) | 18 | 38 | 62 | 31 |
| **% of simulated cells** | **18** | **38** | **62** | **31** |
| **Experimental Data** | **28** | **40** | **51** | **29** |
