# Supplementary material for: Modelling the Role of UCH-L1 on Protein Aggregation in Age-Related Neurodegeneration
Source: PLoS One. 2010 Oct 6;5(10):e13175. doi: 10.1371/journal.pone.0013175 (PMC2950841; doi:10.1371/journal.pone.0013175)
Supplement: Table S10 — Results for S18Y mutant. Mutant has higher hydrolase activity. (0.03 MB DOC) [file pone.0013175.s012.doc]

**Table S10 Results for S18Y mutant. Mutant has higher hydrolase activity**

|  | Number of “simulated cells” with inclusions | | | |
| --- | --- | --- | --- | --- |
| UCH-L1 expression  (Number of runs) | 4h post PI | 6h post PI | 8h post PI | No PI |
| 3x baseline (25) | 18 | 24 | 25 | 21 |
| 2x baseline (50) | 5 | 19 | 29 | 8 |
| Baseline (25) | 0 | 0 | 3 | 0 |
| Total (100) | 23 | 43 | 57 | 29 |
| **% of simulated cells** | **23** | **43** | **57** | **29** |
| **Experimental Data** | **31** | **34** | **48** | **21** |
